# Genome-wide CRISPR-Cas9 knockout screens identify DNMT1 as a druggable dependency in sonic hedgehog medulloblastoma

**DOI:** 10.1186/s40478-024-01831-x

**Published:** 2024-08-07

**Authors:** Foteini Tsiami, Chiara Lago, Noemi Pozza, Federica Piccioni, Xuesong Zhao, Fabienne Lülsberg, David E. Root, Luca Tiberi, Marcel Kool, Jens Schittenhelm, Pratiti Bandopadhayay, Rosalind A. Segal, Ghazaleh Tabatabai, Daniel J. Merk

**Affiliations:** 1 Department of Neurology and Interdisciplinary Neuro-Oncology, Hertie Institute for Clinical Brain Research, University Hospital Tübingen, Eberhard Karls University, Tübingen, Germany; 2 Armenise-Harvard Laboratory of Brain Disorders and Cancer, CIBIO, Trento, Italy; 3 Genetic Perturbation Platform, Broad Institute of MIT and Harvard, Cambridge, MA USA; 4 Merck Research Laboratories, Cambridge, MA USA; 5 Department of Cancer Biology, Dana-Farber Cancer Institute, Boston, MA USA; 6 Department of Neurobiology, Harvard Medical School, Boston, MA USA; 7 Institute for Anatomy, Anatomy and Cell Biology, Rheinische Friedrich-Wilhelms-University, Bonn, Germany; 8 Hopp Children’s Cancer Center (KITZ), Heidelberg, Germany; 9 Division of Pediatric Neurooncology, German Cancer Research Center (DKFZ) and German Cancer Research Consortium (DKTK), Heidelberg, Germany; 10 Princess Máxima Center for Pediatric Oncology, Utrecht, the Netherlands; 11 University Medical Center Utrecht, Utrecht, the Netherlands; 12 Department of Pathology and Neuropathology, Institute of Neuropathology, University Hospital Tübingen, Eberhard Karls University, Tübingen, Germany; 13 Comprehensive Cancer Center Tübingen Stuttgart, University Hospital Tübingen, Eberhard Karls University, Tübingen, Germany; 14 Dana-Farber/Boston Children´S Cancer and Blood Disorders Center, Boston, MA USA; 15 Broad Institute of MIT and Harvard, Cambridge, MA USA; 16 Department of Pediatrics, Harvard Medical School, Boston, MA USA; 17 Cluster of Excellence iFIT (EXC 2180) “Image Guided and Functionally Instructed Tumor Therapies”, Eberhard Karls University, Tübingen, Germany; 18 German Cancer Research Center (DKFZ), German Consortium for Translational Cancer Research (DKTK), Partner Site Tübingen, Heidelberg, Germany

**Keywords:** Medulloblastoma, Functional genomics, DNMT1, SHH pathway, Combinatorial treatment, Synergy

## Abstract

Sonic hedgehog subgroup of medulloblastoma (SHH-MB) is characterized by aberrant activation of the SHH signaling pathway. An inhibition of the positive SHH regulator *Smoothened* (SMO) has demonstrated promising clinical efficacy. Yet, primary and acquired resistance to SMO inhibitors limit their efficacy. An understanding of underlying molecular mechanisms of resistance to therapy is warranted to bridge this unmet need. Here, we make use of genome-wide CRISPR-Cas9 knockout screens in murine SMB21 and human DAOY cells, in order to unravel genetic dependencies and drug-related genetic interactors that could serve as alternative therapeutic targets for SHH-MB. Our screens reinforce SMB21 cells as a faithful model system for SHH-MB, as opposed to DAOY cells, and identify members of the epigenetic machinery including DNA methyltransferase 1 (DNMT1) as druggable targets in SHH-dependent tumors. We show that *Dnmt1* plays a crucial role in normal murine cerebellar development and is required for SHH-MB growth in vivo. Additionally, DNMT1 pharmacological inhibition alone and in combination with SMO inhibition effectively inhibits tumor growth in murine and human SHH-MB cell models and prolongs survival of SHH-MB mouse models by inhibiting SHH signaling output downstream of SMO. In conclusion, our data highlight the potential of inhibiting epigenetic regulators as a novel therapeutic avenue in SMO-inhibitor sensitive as well as resistant SHH-MBs.

**Supplementary Information:**

The online version contains supplementary material available at 10.1186/s40478-024-01831-x.

## Introduction

Embryonal brain tumors comprise a heterogeneous group of undifferentiated or poorly differentiated neuroepithelial tumors of the central nervous system (CNS). Medulloblastoma (MB) is one of the most common malignant pediatric brain tumors that accounts for approximately 70% of all embryonal CNS tumors in the age group of 0–19 years [47]. Standard-of-care therapy including surgical resection of the tumor, cytotoxic chemotherapy, and cranio-spinal irradiation for non-infants, can cure 70–80% of MB patients [53]. However, intensive treatment may induce several long-term side effects. MB is a highly heterogeneous tumor entity that is currently categorized into four main subgroups based on genetic, transcriptomic, and proteomic features: wingless (WNT), sonic hedgehog (SHH), Group 3 and Group 4 [1, 59].

The SHH subgroup accounts for approximately for 30% of all MBs and has a bimodal age distribution, as it appears most often in infants (0–3 years) and adults (> 16 years) but much less frequently in children (3–16 years) [59]. Gene expression and methylome analysis has revealed intratumoral heterogeneity, further stratifying SHH subgroup into four molecular subtypes with distinct clinicopathological and genomic characteristics [36]. SHH-MB patients harbor mutations and copy number variations in major components of the SHH signaling pathway that lead to constitutive activation of the pathway. Mouse models that recapitulate SHH-MB development are well-established and suggest cerebellar granule neuron precursors (GCNPs) as the cell of origin for this subgroup of MB [12, 55]. Targeting SMO has emerged as a targeted treatment option for SHH-driven cancers including basal cell carcinoma [56] and recurrent SHH-MB [51, 52], demonstrating anti-tumor activity by suppressing SHH signaling. In contrast to SHH-MB patients with mutations in *PTCH1*, patients with mutations in downstream components of the SHH pathway, such as loss-of-function mutations of *SUFU* or *GLI2* amplifications which are prevalent in infants and children, are primarily resistant to SMO inhibition [28, 31]. In addition, patients with an initial response to SMO inhibition are prone to develop secondary resistance mechanisms to treatment [29, 52, 63]. In light of these primary or secondary resistance mechanisms, novel therapeutic treatment options are urgently needed that will be efficacious in SHH-MB irrespective of the genetic alterations within the SHH pathway.

Functional genomic screening using the CRISPR-Cas9 system has emerged as a powerful approach to associate genetic perturbations with a distinct phenotype in cancer cells [57]. In particular, genome-wide loss-of-function screens in established cancer cell models allow for the identification of genetic dependencies, thereby prioritizing candidate therapeutic targets [2]. Moreover, CRISPR-based knockout screens are a novel approach to unravel chemogenetic interactors for drugs of interest, leading to synthetic lethal drug target discovery in in vitro model systems [19]. However, while there is an abundance of described Group3/4 MB cell lines, model systems for SHH-MB are scarce, with human DAOY cells being the most cited cell line [23]. In addition, it has been reported that cultured SHH-MB cells do not faithfully recapitulate the activation of the SHH pathway in primary tumors [54], calling into question the reliability of these model systems.

We here performed CRISPR-Cas9 knockout screens in model systems of SHH-MB in order to unravel genetic vulnerabilities on a genome scale. These screens identified murine SMB21 cells as a functionally-relevant model system for SHH-MB. We identified *Dnmt1* as a druggable dependency in SHH-MB and show that DNMT1 inhibition can effectively inhibit tumor growth both in in vitro and in vivo models of SHH-MB by suppressing SHH signaling output. Additionally, by employing chemogenetic CRISPR-Cas9 screens, we discovered that SMO inhibition acts synergistically with DNMT1 inhibition in suppressing tumor proliferation in murine as well as PDOX (patient-derived xenografts organoids) SHH-MB models. Our data propose novel therapeutic strategies for SHH-MB involving DNMT1 inhibition, as these are expected to be efficacious both in SMO inhibitor-sensitive as well as resistant SHH-MB.

## Materials & methods

### Cell lines

SMB21, SMB55 and SMB56 cell lines were previously derived from spontaneous MB tumors from three individual *Ptch*^*+/-*^ mice and show uniform GCNP lineage marker expression as well as aberrant SHH activation [65]. SMB21 cells with loss of *Sufu* and *Gli2* amplification have been previously described as SMO-inhibition resistant cell models [64, 65]. All SMB cells were cultured as neurospheres in ultra-low attachment culture flasks (Corning) with Dulbecco´s Modified Eagle´s Medium/Ham´s F-12 50/50 Mix medium (DMEM/F12) (Corning) supplemented with 2% B27 and vitamin A (Thermo Fisher Scientific), 1% GlutaMax (Thermo Fisher Scientific) and 1% penicillin–streptomycin (Thermo Fisher Scientific). Human pediatric MB cell line DAOY was cultured in Roswell Memorial Institute Medium (RPMI) (Thermo Fisher Scientific) supplemented with 10% fetal calf serum (Thermo Fisher Scientific) and 50µg/ml gentamycin (Thermo Fisher Scientific). For all cell lines, the optimal cell density was determined in order to achieve optimal growing conditions. 200,000 cells per ml was the optimal seeding density for SMB cells and 5,000 cells per cm^2^ for DAOY cells. All cell lines were kept at 37°C humidity-controlled incubator with 5% CO_2_ and regularly tested for mycoplasma contamination.

### Patient-derived xenografts organoids generation and maintenance

Patient-derived xenografts organoids (PDXOs) have been generated and maintained, as previously described [30]. Briefly, PDXOs were maintained in patient-derived organoids medium (PDOs medium) containing 1:1 Neurobasal (Gibco, 21,103,049):DMEM/F12 (Gibco, 11,320,074), 50X B27 supplement (Gibco, 17,504,044), 100X GlutaMax (Gibco, 35,050,038), 100X N2 supplement (Gibco, 17,502,001), 20 ng/ml FGF2 (Peprotech, 100-18B), 20 ng/ml EGF (Peprotech, 100–47), penicillin (100 U/ml)/streptomycin (100 μg/ml) (Gibco, 15,140,122), and Heparin 2.5 μg/ml (Sigma Aldrich, H3149-10KU). PDXOs were cultured in 6-cm/10-cm plates (Sarstedt, 82.1194.500, 82.1472.001) in suspension in PDOs medium on an orbital shaker (70 rpm) placed in a 37°C, 5% CO2 incubator. Twice per week a complete medium change was performed. All PDXOs cultures were regularly tested and confirmed free of Mycoplasma.

### Animals

*Math1-cre* [41], *Math1CreER*^*T2*^ [39﻿], *SmoM2-YFP*^*Fl/Fl*^ [40] mice were obtained from Ulrich Schüller, University Hospital Hamburg, Germany. *Dnmt1*^*Fl/Fl*^ [24] mice were obtained from Rudolph Jänisch, Whitehead Institute for Biomedical Research, Cambridge, USA. Genotyping was performed by PCR using genomic DNA from ear samples. All mice were maintained on a 12-h dark/light cycle and animals of both sexes were used for all experiments.

### CRISPR-Cas9 knockout dependency screens

Cas9-expressing SMB21 cells were screened with the guide-only Brie library, which delivers 78,637 different gRNAs targeting 19,674 murine genes [8], and DAOY cells were screened with a corresponding human library, all-in-one Brunello, which provides 76,441 gRNAs targeting 19,114 human genes [8]. Both cell lines were transduced by spinfection with a predefined viral volume, achieving a MOI (multiplicity of infection) of approximately 0.3. 24 h post transduction, cells were split into technical triplicates with cell numbers estimated to reach a 500 × library coverage, meaning that each gRNA would be present on average in 500 cells. Following puromycin selection for 5 days, cells were propagated in culture for 21 days. To ensure proper transduction rate during the screening, an in-line assay was performed in parallel. At the last day of the screen, genomic DNA was extracted from the remaining cells using the QIAamp DNA Blood Maxi Kit (QIAGEN) from which the integrated sgRNA sequences were PCR-amplified and subjected to Next Generation Sequencing at the Broad Institute at MIT (Cambridge, USA).

### CRISPR-Cas9 chemogenetic screens

Prior to the screen, SMB21 cells were transduced with the lentiviral vector lentiCas9-Blast (Addgene #52,962) and selected with blasticidin, in order to generate SMB21-Cas9 expressing cells, as validated via immunoblots. Similar to the dependency screen, cells were transduced by spinfection with a predetermined volume of the Brie library (#73,633, Addgene) with a MOI of approximately 0.3. Next day, cells were selected with puromycin for 5 days and in-line assay was conducted in parallel to ensure transduction efficiency. 7 days post transduction, cells were split into either DMSO or 5-azacytidine drug arm in duplicates at a 500 × library coverage. Applied 5-azacytidine concentration had been previously optimized, for it to have a cytostatic effect. Following 2 weeks of DMSO control and drug treatment, genomic DNA was isolated from the surviving cells using the QIAamp DNA Blood Maxi Kit (QIAGEN) from which the sgRNA sequences were PCR-amplified and sequenced with next generation sequencing at the Broad Institute (Cambridge, USA).

### CRISPR-Cas9 dependency and drug screens analysis

To account for gene-independent cell responses to CRISPR-Cas9 targeting, we used *CRISPRcleanR*, which corrects sgRNA log_2_ fold changes (LFC) in an unsupervised manner [22]. For the downstream comparative analysis of the two screens, murine gene names were converted to homologous human gene names. For direct comparability of screens from human and murine descent, dependency scores were generated by scaling the corrected LFCs on the basis of pan-species non-essential and pan-essential control genes. Furthermore, we followed two distinct statistical approaches, in order to identify cell-specific essential genes. We used MAGeCK RRA algorithm to identify negatively selected genes [34], as well as supervised BAGEL2 algorithm, which calculates the likelihood that one gene belongs to essential or non-essential class [27]. Shared genes from both methods at FDR < 5% are considered essentials for each screen. Comparative gene enrichment analysis of both screens was performed using *clusterProfiler* R package. For the drug screen, we compared the sgRNA distribution of 5-azacytidine drug arm to either DMSO control arm or reference plasmid using MAGeCK MLE algorithm, which calculates β-scores indicative of the degree of selection per gene [33]. Screen results were visualized using *MAGeCKFlute* R package.

### DNA methylation profiling

SMB21 and SMB55 cells were seeded at their optimal density and treated the next day with 3µM and 5µM 5-azacytidine, respectively, and DMSO control for 24 h. DNA was extracted from cells using QIAamp DNA Blood Mini Kit (QIAGEN). Methylation profiling was performed using Infinium™ Mouse Methylation BeadChip according to the manufacturer´s instructions in the Microarray Unit in DKFZ (Heidelberg, Germany). Differential methylation analysis was performed using the *SeSAMe* R package [67]. To define significantly hypo- and hypermethylated probes, we set a threshold of |β value|≥ 0.1 and p_adjusted value_ < 0.05. Gene enrichment analysis using gene ontology terms was conducted in ShinyGO 0.77.

### RNA sequencing

SMB21 cells were seeded at their optimal density and treated the next day with 3 µM 5-azacytidine for 2 and 48 h, as well as with DMSO control for 48 h. RNA was isolated form cells using the RNeasy Plus Mini Kit (Qiagen). For RNA sequencing, mRNA fraction was enriched using polyA capture from 200 ng of total RNA using the NEBNext Poly(A) mRNA Magnetic Isolation Module (NEB). Next, mRNA libraries were prepared using the NEB Next Ultra II Directional RNA Library Prep Kit for Illumina (NEB) according to the manufacturer’s instructions. The libraries were sequenced as paired-end 50 bp reads on an Illumina NovaSeq6000 (Illumina) with a sequencing depth of approximately 25 million clusters per sample. RNA raw data QC and processing was performed using megSAP (version 0.2–135-gd002274) combined with ngs-bits package (version 2019_11-42-gflb98e63). Reads were aligned using STAR v2.7.3a. Further details can be found under https://nf-core.re/rnaseq. Differential gene expression analysis was performed using *DESeq2* R package [37]. To define significantly differentiated expressed genes (DEG), we set a threshold of |log_2_fold change|≥ 0.58 and p_adjusted value_ < 0.05. All heatmaps were visualized on Morpheus (Broad Institute). For Gene Set enrichment analysis (GSEA), we used the GSEAPreranked tool (Broad Institute), for which genes were ranked based on their log_2_fold change values, according to which gene sets with a false discovery rate (FDR) of less than 25% were considered significantly enriched in our analysis.

### Growth rate inhibition assays

For the acute cytotoxic assays, cells were seeded in 50µl per well at their optimal cell density in 96-well plates. Serial dilutions of the tested drugs were generated and 50µl of concentrated drug was added into the cells 24 h post seeding, corresponding to final concentrations ranging from 0.01nM to 50µM. DMSO-treated wells were used to normalize data. After 72 h, cell viability was measured using CellTiter 96 Aqueous One Solution (Promega) on plate reader GloMax (Promega). To account for differences in division times among different cell lines, drug response was calculated as normalized growth rate inhibition (GR), assessing the potency and efficacy of the tested drugs, using *GRmetrics* R package [16]. The compounds tested, sonidegib, vismodegib and 5-azacytidine, were purchased from Selleckchem and diluted according to manufacturer´s instructions. GR values range from -1 to 1, with -1 to 0 indicating cell death, equal to 0 cytostatic drug effect and 0 to 1 partial growth inhibition. GR_50_ values refer to the concentration at which cell count is half of the control.

### Cell proliferation assays

For the 8-day drug cell proliferation assays, cells were seeded at their optimal cell density in triplicate wells of 6-well plates and treated with corresponding monotherapies or drug combinations. Cell numbers were determined on day 4 and day 8 post seeding. DMSO-treated cells were used as a control.

### Synergy assays

For the cytotoxic synergy assays between 2 drugs, cells were seeded in 50µl per well at their optimal cell density in 96-well plates. Next day, cells were treated with 4 different concentrations of single drugs and 16 concentration combinations of both drugs. DMSO-treated wells were used to normalize data. Following 72 h treatment, cell viability was measured using CellTiter 96 Aqueous One Solution on GloMax. Synergistic scores of the drug combinations were calculated based on zero interaction potency model (ZIP) using *synergyfinder* R package [66].

### PDXOs treatment assay

For the drug treatment experiment, PDXOs (SHH MB—MED1712 and G3 MB—HT0pGF1 [30]) were cultured for 7 days in PDOs medium added with the following drugs: 10µM 5-azacytidine, 1µM sonidegib and 10µM 5-azacytidine + 1µM sonidegib. DMSO-treated PDXOs were used as a control. PDXOs were kept in Ibidi uncoated 96-well black µ-plates (Ibidi, 89,621) placed in a 37°C, 5% CO2 incubator. A complete change medium was performed every 48h for all drug conditions.

### Genetic validation in vitro and in vivo

In order to generate knockdowns of *Smo* and *Dnmt1*, one sgRNA per gene was cloned into lentiCRISPRv2 puro vector (#98,290, Addgene) according to the manufacturer´s protocol. sgRNA sequence for *sgSmo* is 5´-CACCGGAACTCCAATCGCTACCCTG-3´: and for *sgDnmt1*: 5´-CACCGACCTCGGGCCAATCAATCAG-3´. Lentivirus was produced in HEK293FT cells and SMB21, SMB55 and SMB56 cells were transduced by spinfection. Following puromycin selection for 3 days, transduced cells were seeded in 96-well plates and viability was determined after 3 and 7 days post seeding, using CellTiter Blue. Viability was normalized to parental cells.

*Dnmt1*^*Fl/Fl*^ mice [24] were crossed with *Math1-cre::Dnmt1*^*Fl/*+^ mice, in order to generate mice of genetic background *Math1-cre::Dnmt1*^*Fl/Fl*^ and *Math1-cre::Dnmt1*^*Fl/*+^. These animals were sacrificed at p5 and p21, while *Math1-cre* mice were used a wildtype control. To investigate *Dnmt1* ablation during SHH-MB development, *Math1-cre::Dnmt1*^*Fl/*+^ mice were crossed with *Dnmt1*^*Fl/Fl*^*::SmoM2-YFP*^*Fl/Fl*^ mice, resulting in *Math1-cre::Dnmt1*^*Fl/Fl*^*::SmoM2-YFP*^*Fl/*+^ and *Math1-cre::Dnmt1*^*Fl/*+^*::SmoM2-YFP*^*Fl/*+^ mice. Animals were either sacrificed at p5 or monitored for clinical symptoms and sacrificed when manifesting neurological symptoms. *Math1-cre::SmoM2-YFP*^*Fl/*+^ mice were used as a tumor control group. All animal experimental procedures were approved by the regional council of Tuebingen and conducted according to animal welfare regulations (N10-21G license).

### Western blotting

Proteins lysates were extracted from cells and murine tumor tissue using Pierce RIPA buffer (Thermo Fisher Scientific) with phosphatase inhibitors cocktail (1:100) (#5870, Cell Signaling) and sonicated, in order to ensure DNA shearing. 40µg of protein lysates were mixed with 4 × LDS sample buffer and incubated for 10 min at 70°C. Samples were separated electrophoretically in 4–12% NuPage precast gels (Thermo Fisher Scientific) and blotted on nitrocellulose membranes. After blocking the membranes with 5% nonfat dry milk-TBST buffer for 1 h at RT, they were probed with primary antibodies overnight and next day, they were incubated with goat anti-rabbit (1:5000, ab97051, abcam) or goat anti-mouse (1:5000, ab97023, abcam) horse radish peroxidase (HRP)-conjugated secondary antibodies. Proteins were visualized using SuperSignal West Dura solution (Thermo Fisher Scientific) and images were acquired in ChemiDoc imaging machine (BioRad). The following primary antibodies were used at indicated dilutions: GLI1 (1:1000, #2534, Cell Signaling), β-tubulin (1:1000, #86,298, Cell Signaling) and PCNA (1:1000, #2586, Cell Signaling). GLI1 protein bands were quantified using ImageJ by normalizing to corresponding β-tubulin levels of each sample.

### Mouse treatment study

*Math1-creER*^*T2*^ mice were first mated with *SmoM2-YFP*^*Fl/Fl*^ mice, in order to generate mice of genetic background *Math1-creER*^*T2*^*::SmoM2*^*Fl/*+^. For the induction of Cre activity, pups were injected intraperitoneally (i.p.) at postnatal day 5 (P5) with 1mg tamoxifen (T5648-1G, Sigma) dissolved in corn oil (Sigma-Aldrich). Starting at P50, mice were randomized into vehicle control and drug treatment groups. Mice were treated 5 days a week for 3 weeks consecutively with either vehicle control (i.p.), 2.5mg/kg 5-azacytidine monotherapy (i.p.), 20mg/kg sonidegib monotherapy (oral gavage) or combination of both drugs. 5-azacytidine (Selleckchem) was dissolved in 5% DMSO and 30% PEG, sonidegib (Selleckchem) was dissolved in 2% DMSO and 98% corn oil, while vehicle control was diluted in 5% DMSO and 30% PEG300 (Selleckchem). Mice were monitored for clinical symptoms according to a stringent scoring sheet and when they exhibited neurological symptoms, they were sacrificed by transcardiac perfusion. All animal experimental procedures were approved by the regional council of Tuebingen and conducted according to animal welfare regulations (N10-21G license).

### Histological and Immunohistochemical analysis

For histological analysis of mouse experiments, dissected brains were cut in the midline, snap-frozen in liquid nitrogen, fixed in Tissue-Tek medium (O.C.T, Sakura Finetek) and half-brains were sectioned sagittally at 8 µm thickness using a cryotome (LEICA CM 3050 S). For Hematoxylin and Eosin (H&E) staining, sections were fixed with acetone (− 20 °C) and 80% methanol (4°C), washed with PBS and stained with 0.1% hematoxylin (SIGMA-ALDRICH) for 10 min. Following counterstaining with 1% eosin (Care Roth) for 2 min, slides were passed through a graded series of ethanol. Luxol fast blue (LFB) staining was performed in the Institute for Pathology and Neuropathology (Tübingen, Germany). Briefly, sections were fixed in 4.5% formalin for 5 min, incubated o/n in LFB staining solution (~ 55°C) and countersrained with 0.1% cresyl violet.

Immunohistochemistry of all murine brains was performed by fixing sections either in 4% PFA (RT) or acetone (-20°C) and 80% methanol (4°C), depending on the antibody used. Slides were blocked with 10% BSA in PBS-Tween 0.3% for 1 h and incubated with primary antibodies diluted in 2% BSA in PBS-Tween 0.06% o/n at 4°C. The primary antibodies used are Ki67 (1:100, ab16667, abcam), Pax6 (1:400, ab19045, abcam), NeuN (1:400, #24,307, Cell Signaling), Cleaved Caspase 3 (1:100, #9664, Cell Signaling), Dnmt1 (1:200, ab19905, abcam), Dnmt1 (1:100, #5032, Cell Signaling), Dnmt1 (1:200, #MA5-32,547, Thermo Fisher), Dnmt3a (1:100, #3598, Cell Signaling) and Dnmt3b (1:200, #ab2851, abcam). Next day, sections were incubated with horse anti-rabbit (H + L, BA-1100, Vector Laboratories) or goat anti-mouse (H + L, BA-9200, Vector Laboratories) IgG secondary biotinylated antibodies diluted at 1:400 in 2% BSA in PBS-Tween 0.06% for 1 h at RT, followed by a 30-min incubation with avidin/biotin-based peroxidase solution (VECTASTAIN Elite ABC, Vector Laboratories) and staining with NovaRed Substrate-HRP solution (Vector Laboratories) for 1–5 min. Finally, all sections were counterstained with Hematoxylin for 45 s and dehydrated with graded ethanol.

Images were acquired using bright-field microscopy (Zeiss, Axioplan 2) and analyzed on Adobe Photoshop CS5.1 and Gimp (2.10.34). In order to calculate the percentage of antibody-positive cells, we counted the total number of cells in the region of interest (ROI) and the number of cells stained positive for the marker. ImageJ was used for quantification.

To evaluate relative tumor area of *Math1-creER*^*T2*^*::SmoM2*^*Fl/*+^ mice treated with vehicle control or corresponding drugs, we manually outlined the entire cerebellum and evident tumor region in a total of 10 H&E-stained sections per brain in three mice per treatment group using the freehand selection tool in ImageJ. Percentage of tumor to total cerebellum area was calculated in µm^2^.

PDXOs were fixed in 4% paraformaldehyde in PBS at 4°C overnight, cryoprotected in 30% sucrose in distilled H_2_O at 4°C overnight and embedded in Frozen Section Compound (Leica, 3801480). Frozen PDOs were kept at -20°C until processing. PDXOs cryosections at 20 μm were prepared with a cryostat (Thermo Scientific HM525 NX) on glass slides (Thermofisher Scientific, J1800AMNZ). Slides were stored at -20°C until immunohistology. For immunofluorescence staining of PDXOs, cryosections were treated with a permeabilization solution (PBS supplemented with 3% BSA, Seqens/H2B, 033IDB1000-70; 0.3% Triton™ X100, Sigma-Aldrich, T8787; 5% goat serum, Gibco, 16,210,064) for 1h at room temperature. Primary antibody for Ki67 (Rabbit polyclonal anti-Ki67, 1:500, Abcam, ab15580) was incubated overnight at 4°C in antibody solution (PBS supplemented with 3% BSA, Seqens/H2B, 033IDB1000-70; 0.1% Triton™ X100, Sigma-Aldrich, T8787; 1% goat serum, gibco, 16,210,064) and secondary antibody (Alexa Fluor 546 goat anti-rabbit IgG, 1:500, Thermofisher Scientific, A11035) for 1h at room temperature. Nuclei were counterstained with DAPI 10 mM (Abcam, ab228549). Sections and coverslips (Thermofisher Scientific, 15,747,592) were mounted with permanent mounting medium (Histo-Line laboratories, PMT030).

PDXOs immunohistology images were acquired by either confocal imaging by Leica TCS Sp8 (20X objectives) and Leica Application Suite X software (version 3.5.7.23225) or by Nikon TI2 equipped with spinning disc X-light V2 (10X objective) with NIS Element software (version 5.21.03). For all quantifications of immunohistology, samples being compared were processed in parallel and images were acquired using the same settings and lasers power. For Ki67 quantification, a total number of 9–20 images per sample was used and cells positive for the determined markers were manually quantified using the cell counter function in ImageJ. A specific area of ROI was defined and used across all images, avoiding edges or bad regions of the images. A total number of 600–950 DAPI + cells were counted inside the ROI, equally splitting them across considered images. These DAPI + cells were then checked for the positivity for the marker of interest. Data are presented as mean ± s.e.m. of the percentage of Ki67 + cells/DAPI; each dot represents a ROI/image.

### Statistical analysis

All statistical analyses were performed in GraphPad Prism 9 or R (v4.0.5). To compare three or more groups, we used two-way ANOVA with Tukey´s test for multiple comparisons. For the statistical analysis of all cell quantifications in mouse experiments, Fisher’s exact test was used. For the survival analysis of Kaplan–Meier curves, we used the Log-rank test. Differences are considered significant at *P* < 0.05 unless otherwise specified. Venn diagrams were generated using *Venn Diagram* R package and statistical analysis of intersections is derived from *SuperExactTest* R package. For PDOX experiments, the Shapiro–Wilk test was used to validate the assumption of normality. Statistical significance was then determined using Kruskal–Wallis test with Dunn’s post hoc test for data with non-normal distribution.

## Results

### SMB21 cells are a faithful model for SHH-MB

**Fig. 1:**
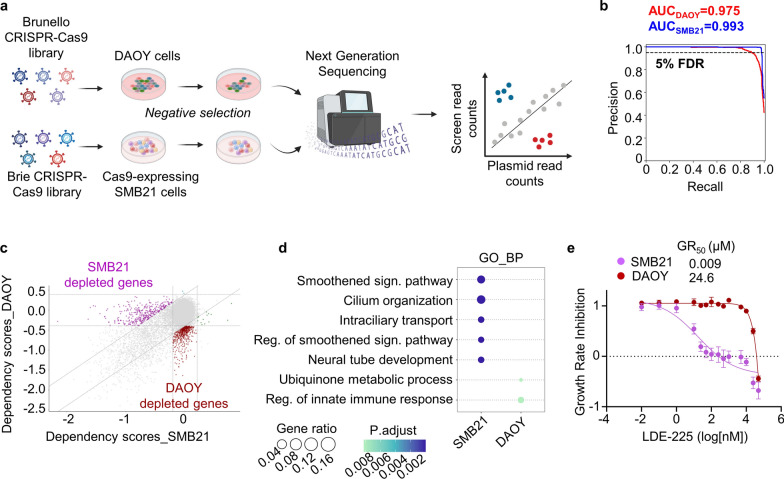
CRISPR-Cas9 knockout screens identify genetic dependencies in DAOY and SMB21 cells. **a** Schematic overview of CRISPR-Cas9 negative selection screening for DAOY and SMB21 cells. **b** Precision-recall curve for knockout screens on the basis of pan-species essential and non-essential genes after CRISPRcleanR correction. **c** 9-square scatter plot demonstrating dependency scores of DAOY and SMB21 cells. ´´Midleft´´ purple data points indicate depleted genes in SMB21 cells, and ´´Bottomcenter´´ red data points depleted genes in DAOY cells. Diagonal dotted lines represent standard deviation of 2. **d** Dotplot illustrating gene ontology analysis of depleted genes identified in SMB21 and DAOY cells using biological process terms (GO_BP). Adjusted *P*-values are color-coded and gene ratio size-coded, as indicated on the side bar. **e** Dose-dependent growth rate inhibition of SMB21 and DAOY cells treated with LDE-225 for 72 h (n = 4). GR_50_ values are indicated for both cell lines. Graph displays mean ± SD

We performed genome-scale CRISPR-Cas9 knockout screens in SMB21 cells, derived from SHH-MB arising in *Ptch*^*+/-*^ mice [65], and human DAOY cells, targeting 19,674 murine and 19,114 human genes, respectively [8] (Fig. 1a). Both screens were analyzed using the same bioinformatic pipeline, including correction for gene-independent effects using *CRISPRcleanR* [22] (Additional file 1: Fig. S1). Precision-recall analyses based on the distribution of pan-species essential and non-essential reference genes revealed good performance of both screens (Fig. 1b). In order to differentiate genetic dependencies between the two cell lines, we directly compared fitness effects in both cell models. Pan-species essential genes were similarly depleted in both screens, while the distribution of non-essentials remained unaffected (Additional file 1: Fig. S2a). However, we observed distinct subsets of depleted genes (345 and 511 genes) in SMB21 and DAOY cells, respectively (Fig. 1c). Gene ontology analyses revealed a significant enrichment of functions related to smoothened signaling pathway and ciliary organization in genes exclusively depleted in SMB21 cells, while none of these terms were enriched in DAOY cells (Fig. 1d; Additional file 1: Fig. S2b; Additional file 2: Table S1). We next defined essential genes in SMB21 and DAOY cells using a combination of MAGeCK-RRA and BAGEL2 algorithms (Additional file 1: Fig. S2c; Additional file 3: Table S2). We identified a significant overlap of shared essential genes between the two cell lines at FDR < 5%, and those genes were mainly associated with essential cellular processes including ribosome biogenesis and RNA processing (Additional file 1: Fig. S2d,e). Functional analysis of SMB21- and DAOY-specific essentialities further revealed significant enrichment of gene sets associated with active SHH signaling in GCNP cells only in the SMB21 line (Additional file 1: Fig. S2f, Additional file 2: Table S1), further suggesting that this cell line represents a functionally-relevant model system for SHH-driven tumors. To further validate the differential dependency of SMB21 and DAOY cells on sonic hedgehog signaling pathway, we evaluated SMO inhibition by treating both cell lines with two distinct SMO inhibitors, Sonidegib (LDE-225) and Vismodegib (GDC-0449) (Fig. 1e; Additional file 1: Fig. S2g). While both SMO inhibitors reduced SMB21 cell viability in a dose-dependent manner, as already shown previously [65], DAOY cells did not respond to SMO inhibition, further corroborating the screen findings that DAOY cells do not depend on SHH pathway for their proliferation and survival. Thus, we only proceeded with screening data from SMB21 cells to identify potential treatment options for SHH-MB.

### DNMT1 is a druggable target in SHH-MB

**Fig. 2:**
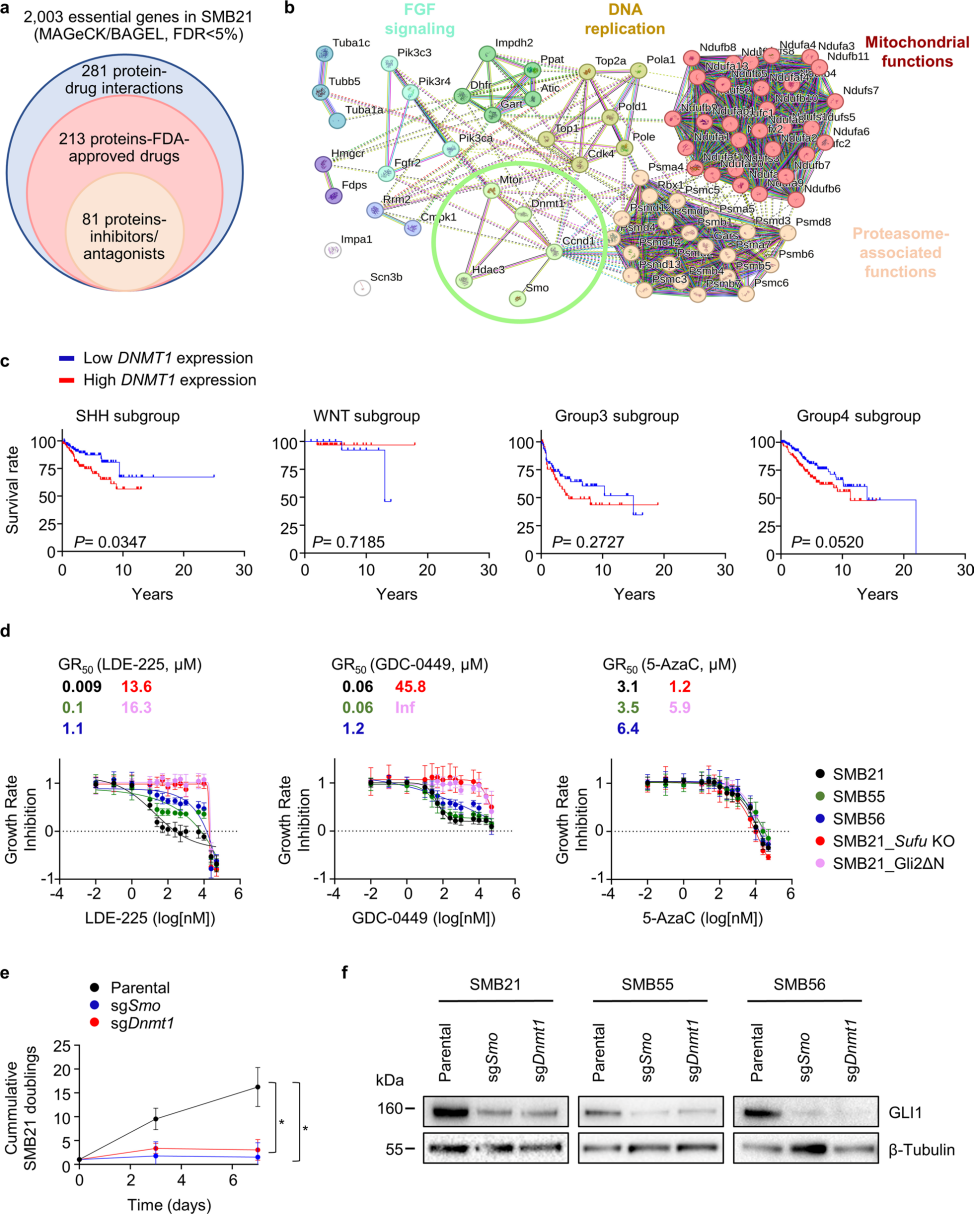
DNMT1 is a druggable dependency in SMB21 cells. **a** Interrogation of SMB21-specific dependency-drug interactions using the Drug-Gene Interaction Database. **b** STRING interaction network depicting physical and functional associations among 81 proteins being targeted by FDA-approved inhibitors or antagonists. Colored nodes indicate different interaction clusters, while white nodes represent genes without any interaction, as determined by MCL clustering. Dashed lines represent inner-cluster edges, while solid lines the type of interaction evidence (STRING database, version 12.0). **c** Survival curve of SHH-, WNT-, Group3- and Group4-MB patients with low and high *DNMT1* expression, using publicly available data [3]. Significance in survival was determined using the log rank (Mantel-Cox) test. **d** Growth rate inhibition curves of parental SMB cells and derivatives of SMB21 cells depicting the efficacy and potency of LDE-225 (left panel), GDC-0449 (middle panel) and 5-AzaC (right panel) for 72 h (n = 4). GR_50_ values are indicated for all cell lines. **e** Analyses of cell population doublings for SMB21 cells transduced with the indicated sgRNA constructs over 7 days. Two-way ANOVA, Tukey´s multiple comparisons test (n = 3). **f** Western blotting illustrating GLI1 protein levels in SMB21, SMB55 and SMB56 control cells and knockout conditions for *Smo* and *Dnmt1*. All graphs display mean ± SD. *p ≤ 0.05

We next investigated potentially druggable targets within SMB21-associated genetic dependencies. After subtracting a list of constitutive core essentials [17], we derived a total of 2,003 essential genes in SMB21 cells, which we interrogated using the Drug-Gene interaction database [6]. We identified 281 genes with predicted drug interactions, out of which 213 genes are targeted by FDA-approved drugs including 81 potential targets for small molecule inhibitors (Fig. 2a). Using the STRING database [58], we generated a protein–protein association network of these 81 druggable dependencies, associating most of these genes with common essential cellular processes such as mitochondrial function and DNA replication (Fig. 2b). Of note, a smaller subcluster of five proteins associated SHH signaling with targetable components of the epigenetic machinery. Out of those, only expression of the DNA methyltransferase 1 (*DNMT1*) served as a prognostic factor in SHH-MB patient data, where high expression correlated with worse overall survival (Fig. 2c; Additional file 1: Fig. S3a) [3]. *DNMT1* expression was not prognostic in any of the remaining MB subgroups. Within different SHH-MB subtypes, γ and δ subtypes are associated with favorable prognosis and lower *DNMT1* expression compared to α and β subtypes (Additional file 1: Fig. S3b). Of note, SHH-α tumors, which are highly enriched for *TP53* mutations and represent a high-risk group with significantly worst prognosis [3, 35, 50], had the highest *DNMT1* expression, and SMB21 cells carry a *TP53* mutation as well [65]. In general, *DNMT1* expression in MB was higher than in corresponding normal tissue and other central nervous system tumors as determined from previous publications (Additional file 1: Fig. S3c) [14, 15].

A previous study has demonstrated the potential of class I HDAC inhibitors to reduce SHH-MB growth by acting downstream of SMO [48], indicating that targeting the epigenetic machinery might be efficacious in tumors resistant to SMO inhibition. We thus aimed to investigate whether targeting the epigenetic regulator DNMT1 using 5-azacytidine (5-AzaC), a hypomethylating agent in clinical use for the treatment of acute myeloid leukemia and myelodysplastic syndromes [7], is efficacious in SHH-MB as well. For validation experiments, we employed distinct SHH-subtype medulloblastoma (SMB) in vitro models derived from *Ptch*^*+/-*^ mice [65], previously shown to uniformly recapitulate SHH-MB hallmark features, as well as cell line derivates of one of those models that incorporate genetic alterations associated with SMO inhibitor resistance [64, 65]. We show that 5-AzaC treatment exerts cytotoxic effects in a dose-dependent manner in all SMB cell lines regardless of the nature of genetic alterations within the SHH pathway, while SMO inhibition proves to be ineffective in the presence of aberrations downstream of SMO (Fig. 2d). Besides pharmacological inhibition, genetic ablation of *Dnmt1* significantly reduced viability of SMB21 cells similar to the loss of *Smo* (Fig. 2e), further corroborating the screening results that *Dnmt1* is required for SHH-MB proliferation. Additionally, both knockout of *Dnmt1 and Smo* strongly decreased GLI1 protein levels in all SMB cell models (Fig. 2f), suggesting that loss of *Dnmt1* affects SHH pathway output. In summary, we show that SHH-MB cells depend on the epigenetic regulator DNMT1 for their survival and therefore, pharmacological inhibition of DNMT1 using 5-AzaC might serve as an efficacious therapeutic strategy for SHH-MB regardless of the nature of genetic aberrations within the SHH pathway.

### Genetic loss of *Dnmt1* affects normal cerebellar and SHH-MB development in vivo

**Fig. 3:**
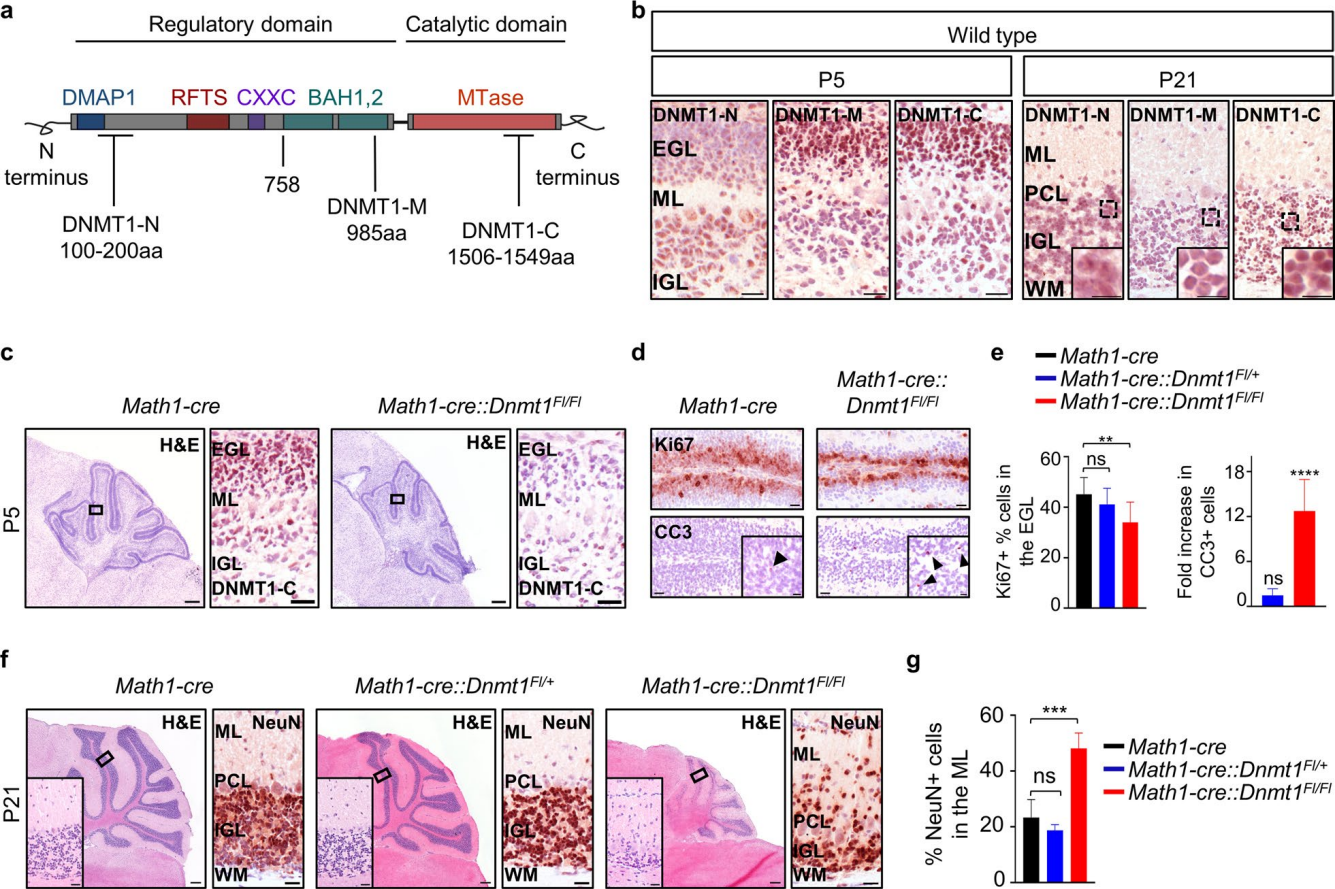
The role of *Dnmt1* in cerebellar granule neuron development. **a** Schematic overview of protein domains for mouse DNA methyltransferase 1 (1620 amino acids), and binding sites for three distinct DNMT1 antibodies (DNMT1-N, DNMT1-M and DNMT1-C). DMAP1, DNMT1 associated protein 1; RFTS, replication foci targeting sequence; CXXC, zinc finger; BAH1/2, bromo adjacent homology domains; MTase, C-5 methyltrasnferase. **b** Representative images of wild type cerebella stained with DNMT1-N, DNMT1-M and DNMT1-C antibodies at P5 and P21. **c** Representative H&E stainings of cerebella from *Math1-cre* and *Math1-cre::Dnmt1*^*Fl/Fl*^ mice at P5, as well as immunohistochemistry for DNMT1-C at indicated magnified region. **d** Immunohistochemistry for Ki67 and Cleaved Caspase 3 (CC3) in the EGL of cerebella from mice with indicated genotypes. Black arrowheads in the inset indicate CC3 positive cells. **e** Quantification of Ki67 positive cells and fold increase of CC3 positive cells compared to *Math1-cre* mice, as shown in (**d**) (n = 4, Fisher´s exact test). **f** Exemplary cerebellar sections of *Math1-cre*, *Math1-cre::Dnmt1*^*Fl/*+^ and *Math1-cre::Dnmt1*^*Fl/Fl*^ mice at P21 mice stained with hematoxylin & eosin, and with antibody against NeuN. **g** Quantification of NeuN-positive cells in the ML of cerebella of mice with indicated genotypes at P21, as shown in (**f**) (n = 3, Fisher´s exact test). 4 × magnification, scale bar, 500µm; 20 × magnification, scale bar, 50µm; 40 × magnification, scale bar, 20µm. EGL, external granular layer; ML, molecular layer; PCL, Purkinje cell layer; IGL, internal granular layer; WM, white matter. All graphs display mean ± SD. ** *p* ≤ 0.01, *** *p* ≤ 0.001, **** *p* ≤ 0.0001

DNMT1 is a maintenance methyltransferase that catalyzes the transfer of a methyl group from S-adenosyl-L-methionine to the 5´ position of cytosine nucleotides. It is known to be critical for embryonal development, as conditional knockout of *Dnmt1* in embryonal stem cells results in abnormal murine development and embryonal lethality [32]. Mammalian DNMT1 consists of an *N*-terminal regulatory domain and a *C*-terminal catalytic domain which is essential for DNA binding and transferring of methyl unit (Fig. 3a) [38], and different isoforms in somatic cells, oocytes and embryos have been identified [11, 45]. Having shown that *Dnmt1* is required for SHH-MB proliferation in vitro, we first sought to evaluate its protein expression patterns in the murine cerebellum. For that, we tested three antibodies that recognize different regions of the full-length DNMT1 protein and assessed signal from immunohistochemistry in wild type mice. Reaction of an DNMT1 antibody binding the N terminus (DNMT1-N) was strongest in mature granule neurons of the internal granular layer (IGL) (Fig. 3b). In contrast, antibodies recognizing the bromo adjacent homology domain (DNMT1-M) or the catalytic domain (DNMT1-C) of the DNMT1 protein showed the strongest signal in proliferating GCNPs of the external granule layer (EGL). All antibodies demonstrated basal expression levels in differentiated neurons of the IGL at P21, corroborating previous studies of *Dnmt1* mRNA and protein expression in the murine cerebellum and cerebellar neurons [9, 13, 21]. Taken together, these data indicate the presence of distinct DNMT1 isoforms during the maturation of cerebellar granule neurons, with the majority of catalytic active DNMT1 being expressed in GCNPs.

In order to address the role of DNMT1 during cerebellar development, we conditionally ablated *Dnmt1* in GCNPs in vivo using the Cre/loxP system under the control of a Math1 promoter [41], resulting in an out-of-frame splice from exon 3 to exon 6 of *Dnmt1*, leading to a truncated mRNA encoding the first 67 amino acids of the protein [24]. Immunostaining using DNMT1-C antibody validated the loss of *Dnmt1* in GCNPs in the cerebella of *Math1-cre::Dnmt1*^*Fl/Fl*^ mice at postnatal day 5 (Fig. 3c). In parallel, we investigated the expression of two de novo DNA methyltransferases, DNMT3A and DNMT3B, which play important roles during early and later in development, respectively [46]. Immunohistochemical analysis revealed that DNMT3A is weakly expressed in proliferating GCNPs in the EGL and stronger in cells of the IGL in Math1-cre mice at P5, while DNMT3B expression is virtually absent in the cerebellum of these mice (Additional file 1: Fig. S4), supporting previous data showing distinct patterns of expression for both enzymes in the CNS [10, 46, 61]. The expression of both enzymes remains unaffected in *Math1-cre::Dnmt1*^*Fl/Fl*^ mice.

Furthermore, immunohistochemical analysis using antibodies against Ki67 and cleaved caspase 3 revealed a significant reduction in proliferating GCNPs and conversely a significant increase in apoptotic GCNPs in the EGL of *Math1-cre::Dnmt1*^*Fl/Fl*^ mice as compared to control mice (Fig. 3d, e). Proliferative and apoptotic activity of GCNPs in mice with a heterozygous loss of *Dnmt1* (*Math1-cre::Dnmt1*^*Fl/*+^) did not statistically differ from wild type mice (Additional file 1: Fig. S5a,b). Furthermore, analysis of cerebella from *Math1-cre::Dnmt1*^*Fl/Fl*^ mice at P21 demonstrated severe cerebellar hypoplasia, a phenotype not observed in *Math1-cre::Dnmt1*^*Fl/*+^ mice (Fig. 3f). While neither proliferative nor apoptotic GCNPs were detected in any of the three groups at P21, we observed a significant increase in NeuN and Pax6 positive cells in the molecular layer in *Math1-cre::Dnmt1*^*Fl/Fl*^ mice, suggesting that these are differentiated granule neurons which had been stalled in their migration to the IGL (Fig. 3f,g; Additional file 1: Fig. S5c,d). Taken together, we show that loss of *Dnmt1* affects normal development of GCNPs, resulting in migrational deficits and cerebellar hypoplasia.

**Fig. 4:**
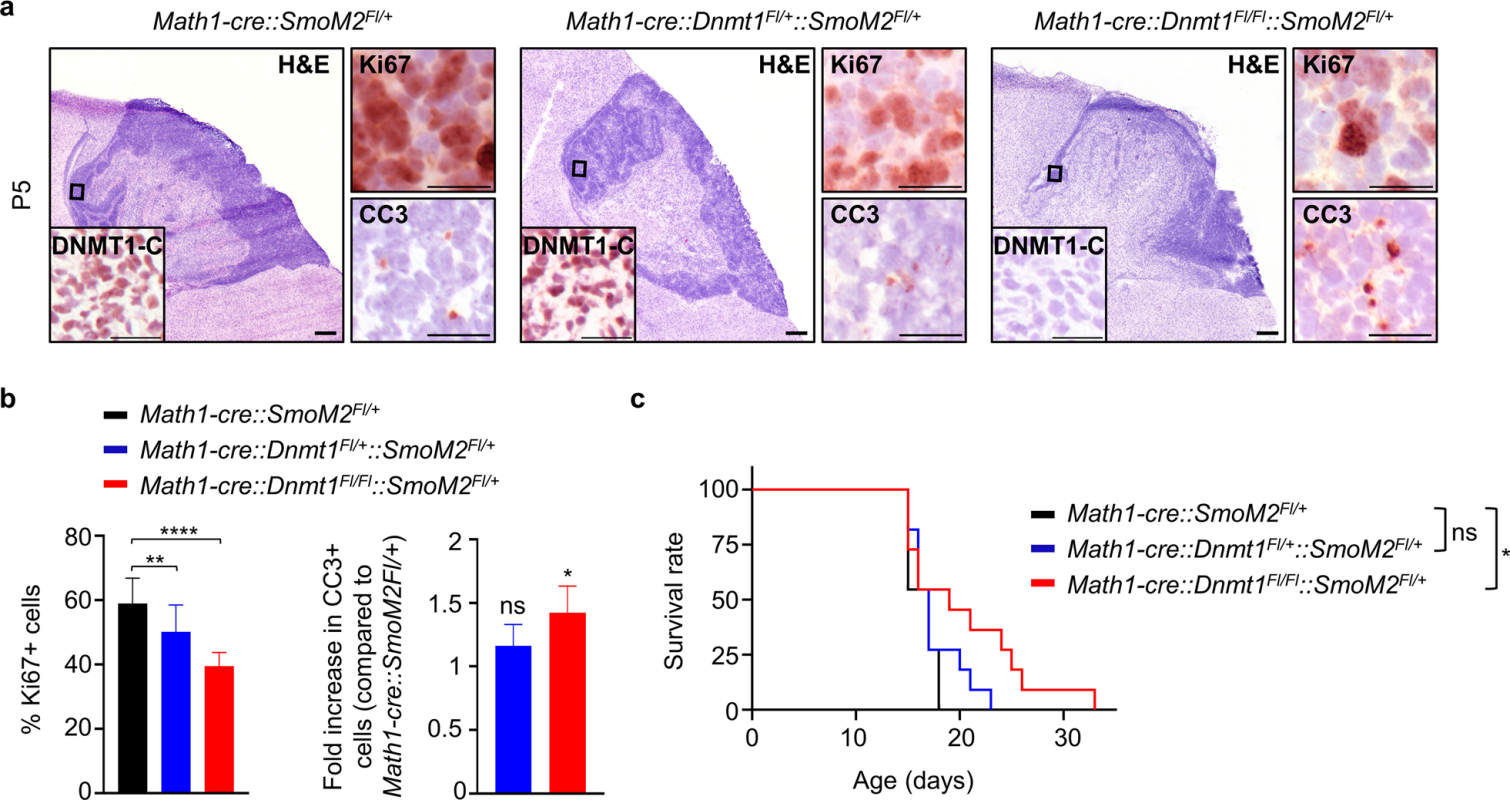
*Dnmt1* genetic loss during SHH-MB development. **a** Representative H&E stainings and immunohistochemistry for DNMT1-C, Ki67, and cleaved Caspase 3 (CC3) in tumors from *Math1-cre::SmoM2*^*Fl/*+^, *Math1-cre::Dnmt1*^*Fl/*+^*::SmoM2*^*Fl/*+^ and *Math1-cre::Dnmt1*^*Fl/Fl*^*::SmoM2*^*Fl/*+^ mice at P5. **b** Quantification of Ki67- (left) and CC3-positive cells (right) in tumors from mice with indicated genotype at P5 (n = 4, Fisher´s exact test). **c** Kaplan–Meier curves of *Math1-cre::SmoM2*^*Fl/*+^ mice (n = 11) compared to heterozygously (n = 11) and homozygously (n = 11) *Dnmt1*-depleted *Math1-cre::SmoM2*^*Fl/*+^ mice. Significance in survival was determined using the log rank (Mantel-Cox) test. 4 × magnification, scale bar, 500µm; 20 × magnification, scale bar, 50µm. All graphs display mean ± SD. * p ≤ 0.05, ** *p* ≤ 0.01, **** *p* ≤ 0.0001

We next investigated the contribution of *Dnmt1* to SHH-MB growth in vivo. Here, we genetically ablated *Dnmt1* in GCNPs in an established mouse model of SHH-MB, *Math1-cre::SmoM2*^*Fl/*+^ mice [40] (Fig. 4a). Both mice with a heterozygous or homozygous loss of *Dnmt1* (*Math1-cre::Dnmt1*^*Fl/*+^*::SmoM2*^*Fl/*+^ and *Math1-cre::Dnmt1*^*Fl/Fl*^*::SmoM2*^*Fl/*+^, respectively) showed a significant reduction in the number of proliferating tumor cells in the cerebellum at P5, as compared to *Math1-cre::SmoM2*^*Fl/*+^ mice (Fig. 4a,b). Additionally, *Math1-cre::Dnmt1*^*Fl/Fl*^*::SmoM2*^*Fl/*+^ mice displayed a significant increase in apoptotic cells in their tumors. In line with these findings, mice with a homozygous loss of *Dnmt1* showed a significantly longer survival when compared to control tumor mice (*P* = 0.0324) (Fig. 4c). Summarizing, we show that *Dnmt1* expression in GCNPs is essential for SHH-MB growth in vivo.

### DNMT1 inhibition affects SHH pathway activation

**Fig. 5:**
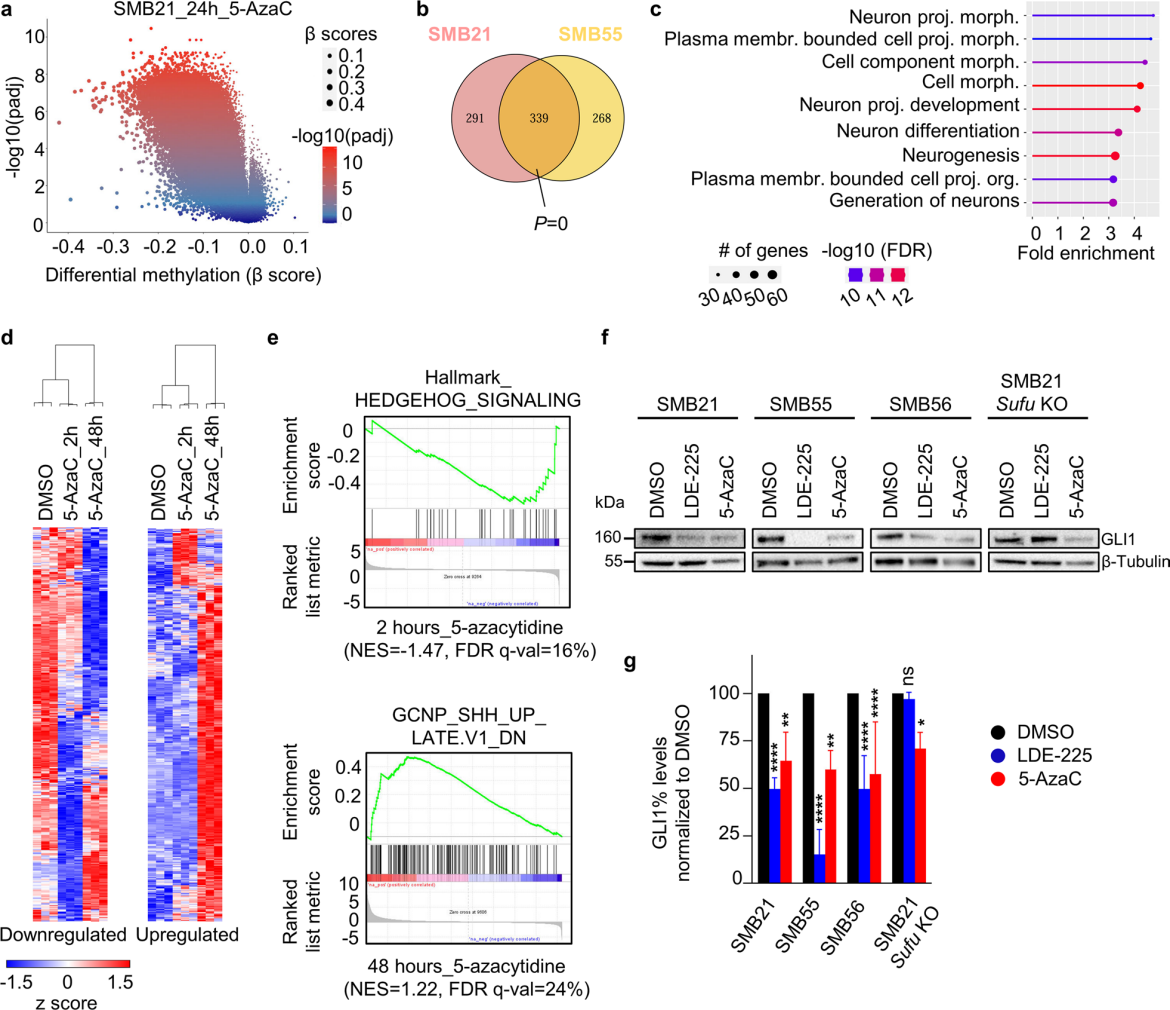
DNMT1 inhibition suppresses SHH-MB tumor cell proliferation by inhibiting SHH signaling pathway. **a** Volcano plot of differentially methylated probes in SMB21 cells treated with 3µM 5-AzaC for 24 h as compared to DMSO control-treated cells. Adjusted *P*-values are color-coded, and β scores are size-coded. **b** Venn diagram illustrating the overlap of genes determined to be significantly hypomethylated in SMB21 and SMB55 cells. Statistics are derived from a *SuperExactTest*. **c** Lollipop plot of the top biological processes of commonly hypomethylated genes between SMB21 and SMB55 cells after 24h treatment of 5-AzaC. **d** Unsupervised hierarchical clustering of significantly downregulated (left) and upregulated (right) genes in SMB21 cells treated with DMSO control or 3µM 5-AzaC for 2 and 48 h (One minus Pearson´s correlation). **e** Gene set enrichment analysis using the Hallmark database revealed a significant downregulation of a gene set associated with active SHH signaling after 2 h of treatment with 5-AzaC (normalized enrichment score NES = -1.47, FDR q-value = 16%, upper panel). Enrichment analysis for C6 oncogenic signature gene sets revealed a significant upregulation of genes known to be inhibited by active SHH signaling in granule cerebellar neuron precursors after 48 h treatment with 5-AzaC (NES = 1.22 and FDR q-value = 24%, lower panel). **f** Representative western blot analyses of GLI1 protein in SMB21, SMB55, SMB56, and SMB21 *Sufu* KO cell lines treated with LDE-225 or 5-AzaC for 48 h. **g** Quantification of GLI1 protein levels in SMB parental and SMB-derivative cells under indicated drug treatments shown in (**f**). Two-way ANOVA, Tukey´s multiple comparison test (n = 3). Graph displays mean ± SD. * p ≤ 0.05, ** *p* ≤ 0.01, **** *p* ≤ 0.0001

We next aimed to investigate changes in global DNA methylation patterns and concomitant changes in gene expression upon DNMT1 inhibition in SHH-MB cells. First, we treated SMB21 and SMB55 cells with their corresponding GR_50_ values of 5-AzaC as determined via growth rate inhibition assays (3µM and 5µM, respectively), or the corresponding DMSO control for 24 h and performed DNA methylation array. We observed a profound hypomethylation upon DNMT1 inhibition in both cell lines (73,594 and 98,794 hypomethylated probes at adjusted *p* < 0.05 in SMB21 and SMB55 cells, respectively) (Fig. 5a; Additional file 1: Fig. S6a; Additional file 4: Table S3). These probes showed a significant enrichment for 630 and 607 genes in SMB21 and SMB55 cells, respectively, displaying a highly significant overlap across both cell models (Fig. 5b). Functional annotation of these shared hypomethylated genes revealed their involvement in fundamental processes of the central nervous system such as neurogenesis and neuronal differentiation (Fig. 5c; Additional file 1: Fig. S6b,c).

Having demonstrated that DNMT1 inhibition alters the DNA methylation pattern in SHH-MB cells, we next performed RNA sequencing of SMB21 cells treated with 3µM 5-AzaC for 2 or 48 h, in order to investigate early and late downstream mechanisms of DNMT1 blockade. Of note, while we exclusively observed hypomethylation in our array analyses, gene expression profiling revealed both up- and down-regulated genes upon DNMT1 inhibition both at 2 and 48 h (Additional file 1: Fig. S6d; Additional file 5: Table S4). Strikingly, we observed strong differences in genes that are deregulated early and late upon drug treatment, with the majority of gene expression changes confined to either early or late stages (Fig. 5d; Additional file 1: Fig. S6e). This suggests that genes which show an early effect are modulated more directly by DNMT1, while late gene expression changes might be governed by gene networks downstream of DNMT1 function.

To gain further insights into the functional consequences of gene expression changes upon DNMT1 inhibition, we performed gene set enrichment analyses (Additional file 6: Table S5). One of the top gene sets downregulated early upon treatment with 5-AzaC was the Hallmark gene set ´Hedgehog signaling´, suggesting that DNMT1 inhibition has an immediate effect on positive regulators of the SHH pathway. In line with this inhibitory effect on SHH signaling, enrichment analysis for late treatment effects revealed a significant upregulation of genes (´GCNP_SHH_UP_LATE_DN´) which are known to be downregulated upon SHH activation in GCNPs, the cell of origin of SHH-MB (Fig. 5e). We next validated SHH pathway blockade by DNMT1 inhibition at the protein level by evaluating GLI1 protein expression. In line with the RNA sequencing findings, we show that 5-AzaC treatment for 48 h significantly reduces GLI1 protein levels in SMB21, SMB55 and SMB56 cells compared to DMSO-treated cells (Fig. 5f,g). Of note, 5-AzaC treatment also significantly reduced GLI1 expression in SMB21 *Sufu* KO cells that are resistant to SMO inhibition. Together, our data indicate that DNMT1 inhibition induces widespread methylation and gene expression changes in SHH-MB. Among other potential effects, these changes reveal a profound suppression of SHH pathway activation in SHH-MB cell lines regardless of the genetic alteration within the SHH signaling pathway.

### Inhibition of DNMT1 and SMO synergistically blocks SHH-MB growth

**Fig. 6:**
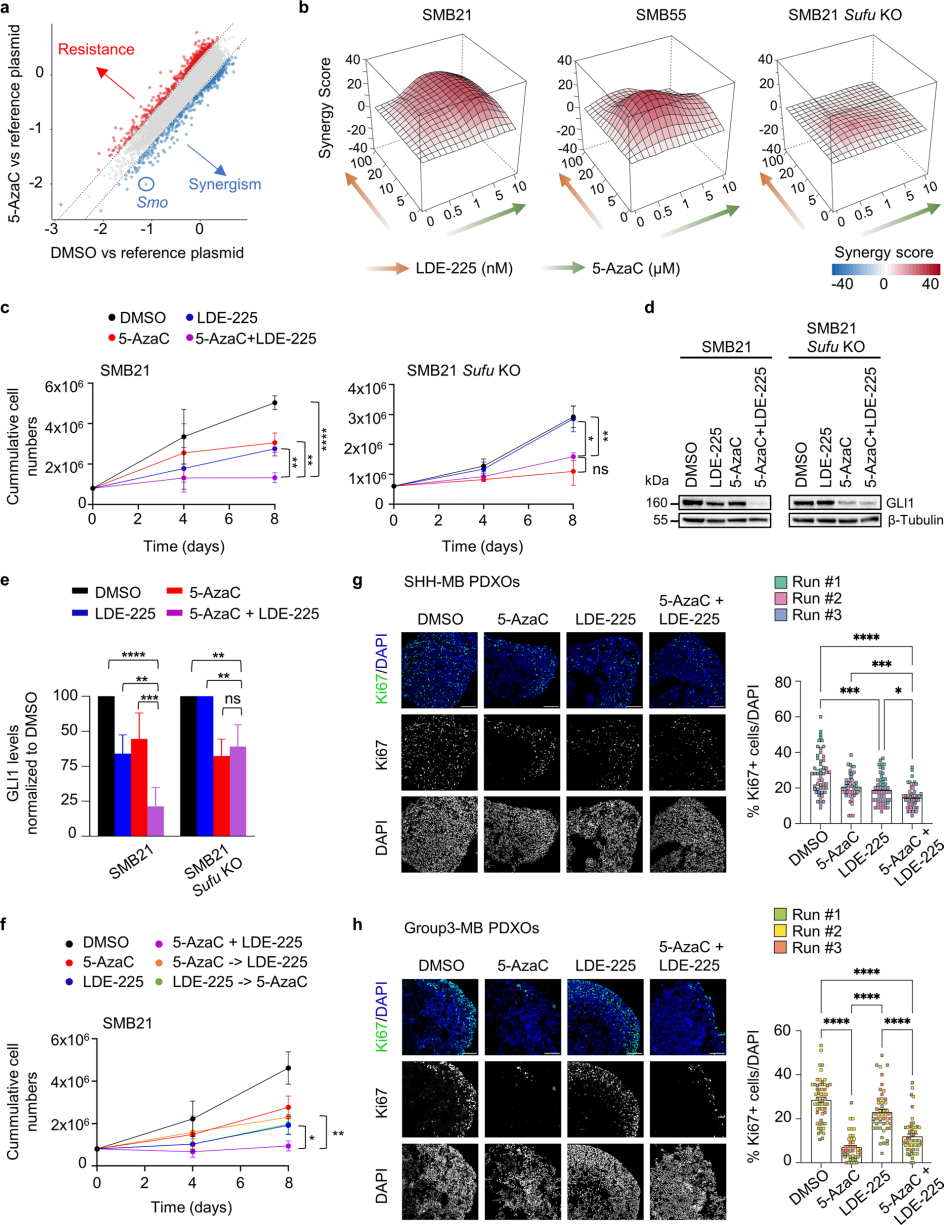
Knockout CRISPR-Cas9 screen unravels loss of *Smoothened* as a synthetic lethal interaction for DNMT1 inhibition. **a** Scatter plot illustrating correlation of β scores of 5-AzaC-treated cells (y axis) and DMSO control-treated cells (x axis) compared to the reference plasmid. Dotted lines represent 1.5-fold standard deviation. **b** 3D interaction landscapes evaluating interaction between LDE-225 and 5-AzaC in SMB21 (left), SMB55 (middle), and SMB21 *Sufu* KO cells (right) (n = 5). Gradient arrows represent concentration range of LDE-225 (orange) and 5-AzaC (green) applied to the cells. Synergism was calculated based on the ZIP model. **c** 8-day proliferation assays of SMB21 (left) and SMB21 *Sufu* KO cells (right) assessing 5-AzaC monotherapy, LDE-225 monotherapy, and drug combination compared to DMSO-treated cells. Two-way ANOVA, Tukey´s multiple comparisons test (n = 4). **d** Western blot analyses of GLI1 protein in SMB21 (left) and SMB21 *Sufu* KO cells (right) treated with indicated drugs for 48 h. **e** GLI1 protein quantification of western blots shown in **d**. Two-way ANOVA, Tukey´s multiple comparisons test (n = 3). **f** 8-day proliferation assays evaluating simultaneous and sequential combinatorial treatment of 5-AzaC and LDE-225 in SMB21 cells. Two-way ANOVA, Tukey´s multiple comparisons test (n = 5). **g** Representative confocal images of PDXOs representing SHH-MB (left). Organoids were treated with indicated drugs (10µM 5-AzaC, 1µM LDE-225, or combination) or DMSO control for 7 days and stained for DAPI and Ki67. Quantification of the fraction of Ki67-positive cells in SHH-MB PDXOs (right). Different replicates are color-coded (n = 3, Kruskal–Wallis test). **h** Same analyses as in (g), employing a Group3-MB PDXO model system. 20 × magnification, scale bar, 150µm. Graphs display mean ± SD (c, e, f) or ± sem (g, h). * p ≤ 0.05, ** *p* ≤ 0.01, *** *p* ≤ 0.001, **** *p* ≤ 0.0001

In light of the potential of DNMT1 to serve as a therapeutic target in SHH-MB, we next aimed to unravel synthetic lethal interactors of DNMT1 inhibition that could serve as potential combinatorial treatment targets in SHH-MB. Thus, we conducted an additional CRISPR-Cas9 knockout screen under 5-AzaC treatment or corresponding DMSO control in SMB21 cells, observing good screening signal as assessed by the depletion of known pan-essential genes in the DMSO arm (Additional file 1: Fig. S7a). In our knockout screen we identified 43 hits at FDR < 10%, out of which *Smo* scored with the highest negative beta score in the drug versus DMSO control comparison, indicating that inhibition of SMO will synergize with 5-AzaC in inhibiting SHH-MB growth (Fig. 6a; Additional file 1: Fig. S7b; Additional file 7: Table S6). Similarly, on the sgRNA level the four different sgRNAs targeting *Smo* had lower counts in the 5-AzaC arm, as compared to the DMSO control arm and the reference plasmid (Additional file 1: Fig. S7c).

To validate these screening results, we assessed combination treatment of LDE-225 and 5-AzaC in a 3-day cytotoxic assay in SMB cells. We observed synergistic interaction between both drugs in SMB21 cells (mean synergy score = 11.7), as well as in SMB55 cells (mean synergy score = 10.46), while no synergism was observed in SMB21 *Sufu* KO cells (mean synergy score = 2.7) (Fig. 6b). Similarly, drug synergy was further validated in SMB21 cells by performing 8-day proliferation assays, showing that combination treatment of LDE-225 and 5-AzaC was significantly more efficacious in inhibiting tumor growth than both monotherapies (Fig. 6c). Again, combination therapy did not differ from the effect of 5-AzaC monotherapy in SMB21 *Sufu* KO cells, and SMO inhibition alone did not block growth of these cells. Using GLI1 protein expression as a surrogate for SHH pathway activation, we also observed synergistic interaction of DNMT1 and SMO inhibitors to block SHH activation in SMB21 cells (Fig. 6d,e). As expected, this synergism was not observed in SMO-inhibitor resistant SMB21 *Sufu* KO cells. Additionally, we sought to answer whether treatment of LDE-225 and 5-AzaC is more potent when combined simultaneously or sequentially. For the sequential treatment, we evaluated LDE-225 treatment followed by 5-AzaC monotherapy treatment, as well as 5-AzaC treatment followed by LDE-225 monotherapy in SMB21 cells (Fig. 6f). Our results demonstrate that both sequential treatments did not differ significantly from the corresponding monotherapies, while simultaneous combination treatment was significantly more efficacious than both of the sequential treatments, suggesting that synergistic actions of LDE-225 and 5-AzaC depend on a simultaneous treatment regimen.

Last, in order to corroborate the translational potential of our findings, we assessed both monotherapies as well as their combination in human patient-derived xenograft organoids (PDXOs) [30] representing SHH- and Group3-subgroup medulloblastoma (Fig. 6g,h). Inhibition of DNMT1 alone effectively blocked proliferation in both models (Fig. 6g,h), being well in line with the finding that DNMT1 represents a genetic dependency in the vast majority of human cancer cell lines (https://depmap.org/portal/). As expected, we found that Group3-MB tumor cells do not respond to SMO inhibition, while SHH-MB cells do. Most importantly, combination treatment in SHH-MB was more efficacious in inhibiting tumor cell proliferation than both monotherapies, while combination treatment in Group3-MB did not show any benefit over DNMT1 inhibition alone. Taken together, these data provide evidence that simultaneous inhibition of DNMT1 and SMO synergistically inhibits SHH-MB tumor growth specifically by blocking SHH pathway output.

### DNMT1 inhibition is efficacious in a SHH-MB mouse model

**Fig. 7:**
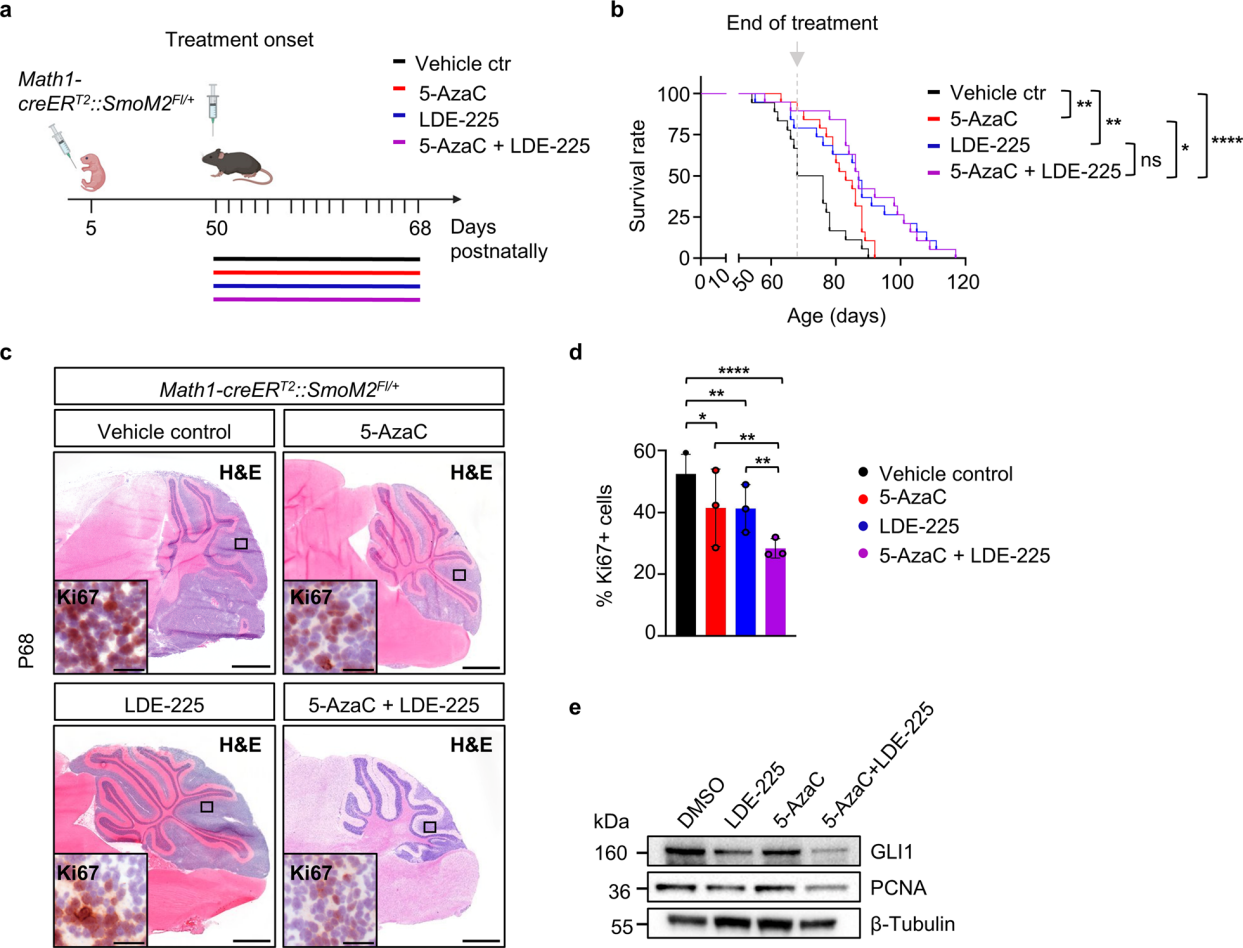
DNMT1 and SMO inhibition synergize to block SHH-MB growth in vivo. **a** Schematic overview illustrating tumor induction and treatment timeline in *Math1-creER*^*T2*^*::SmoM2*^*Fl/*+^ mice. **b** Kaplan–Meier curves of *Math1-creER*^*T2*^*::SmoM2*^*Fl/*+^ mice treated with 5-AzaC monotherapy (n = 19), LDE225 monotherapy (n = 19), drug combination treatment (n = 19), and vehicle control-treated mice (n = 18). Vertical dashed line represents the last day of treatment. Significance in survival as compared to vehicle-treated mice was determined using the log rank (Mantel-Cox) test. **c** Representative H&E stainings of cerebellar tumors from *Math1-creER*^*T2*^*::SmoM2*^*Fl/*+^ mice from indicated treatment groups on the last day of treatment (P68), as well as immunohistochemistry for Ki67 in these tumors. **d** Quantification of Ki67 shown in **c** (n = 3, Fisher´s exact test). **e** Western blot analysis of GLI1 and PCNA proteins deriving from harvested tumor tissue from *Math1-creER*^*T2*^*::SmoM2*^*Fl/*+^ mice at P68. 4 × magnification, scale bar, 500µm; 20 × magnification, scale bar, 50µm. Graph displays mean ± SD. * p ≤ 0.05, ** *p* ≤ 0.01, **** *p* ≤ 0.0001

Finally, we explored the potential of DNMT1 inhibition, alone or in combination with SMO inhibition, as a therapeutic approach in SHH-MB in vivo. In order to ensure a suitable treatment window and minimize potential adverse effects that might arise from treating young postnatal pubs, we made use of a tamoxifen-inducible *Math1-creER*^*T2*^ mouse line [39], thus generating a SHH-MB mouse model that presents delayed mortality at later stages of adulthood [44]. Tumor formation was initiated by tamoxifen injection at P5, and mice were randomized to receive either vehicle control, 5-AzaC monotherapy, LDE-225 monotherapy, or 5-AzaC/LDE-225 combination treatment at P50 (Fig. 7a). Treatment was conducted five days a week for three weeks consecutively, with combination treatment of 5-AzaC and LDE-225 being administered simultaneously, since this was superior to sequential treatment in our in vitro experiments. Kaplan Meier survival analyses revealed that mice treated with 5-AzaC monotherapy and LDE-225 monotherapy had a significant prolonged survival (*P* = 0.0073 and *P* = 0.0014, respectively) compared to vehicle treated mice, with no statistical difference between the monotherapies (*P* = 0.0729) (Fig. 7b). Similarly, mice treated with the combination of both drugs also displayed a significant survival benefit compared to vehicle control group (*P* = 0.0001). However, while the combination therapy had a significantly prolonged survival when compared to the 5-AzaC monotherapy (*P* = 0.0134), it failed to show a significant difference from the LDE-225 monotherapy (*P* = 0.9099). The treatment regimen was terminated for all surviving animals at P68 and therefore did not test whether continued treatment could benefit survival past this timepoint. Thus, in order to assess potential differences among the treatment groups, we included direct comparisons of histology and tumor proliferative status at the last day of treatment. Quantification of relative tumor areas revealed that combination therapy significantly reduced tumor burden in the cerebella of these mice, as compared to both monotherapies and DMSO control (Fig. 7c; Additional file 1: Fig. S8). Furthermore, quantification of Ki67-positive cell fractions revealed a significantly lower proliferation index in tumors from the combination therapy group as compared to both monotherapies (Fig. 7d). Moreover, western blot analyses of tumors from the combination therapy group displayed the strongest decrease in protein levels of proliferating cell nuclear antigen marker PCNA and GLI1 as compared to vehicle control tumors (Fig. 7e). Therefore, we conclude that DNMT1 inhibition is efficacious in inhibiting growth of SHH-MB in vivo. Furthermore, our data indicate that continuous combination therapy of DNMT1 and SMO inhibitors acts synergistically to inhibit tumor growth.

## Discussion

Inhibition of SHH signaling pathway by targeting *Smoothened* constitutes a potential therapeutic option for SHH-driven tumors including medulloblastoma. Although small-molecule SMO inhibitors have exhibited promising anti-tumor activity in SHH-MB, certain mutations within the SHH pathway can limit the efficacy of SMO inhibition, resulting in therapy resistance [28, 31]. Therefore, novel targeted therapies are urgently needed for SHH-MB.

In this study, we first set out to validate the suitability of two distinct SHH-MB cell culture systems, human DAOY and mouse SMB21 cells [25, 65], in order to investigate SHH-associated dependencies using a functional genomics approach. Our data reveal a strong dependency of SMB21 cells on positive SHH regulators such as *Smo* and *Gli2*, and cilia-associated functions which have been known to be required for active SHH transduction [5, 18, 20], while we did not observe any SHH-associated vulnerabilities in DAOY cells. In line with these screening data, SMB21 and other murine SHH-MB derivatives are highly sensitive to SMO inhibition, while DAOY cells proved to be resistant to SHH inhibition as suggested previously [49]. Together, our functional data highlight the potential of murine SHH-MB cell lines to serve as suitable model systems for the investigation of vulnerabilities directly associated with active SHH pathway activation which has previously been shown to be functionally relevant for tumor initiation and growth [12, 52].

Following these initial observations, we next explored the landscape of the targetable proteome of genetic essentialities in SMB21 cells. We identified 81 genes targeted by FDA-approved inhibitors [6], and further protein interaction network analyses [58] suggested functional interaction of the SHH pathway with epigenetic as well as cell cycle-associated regulators including DNMT1 and CCND1. We here suggest DNMT1 as a promising target for therapeutic intervention in SHH-MB based on several observations in our study. First, DNMT1 scored in our dependency screen, and genetic as well as pharmacologic validation both in vitro as well as in vivo supported the essential role of DNMT1 in SHH-MB as well as their SHH-dependent cell of origin. Second, DNMT1 is highly expressed in primary human SHH-MB compared to healthy brain tissue, and high expression correlates with significantly worse patient survival. Third, inhibition of DNMT1 blocked SHH pathway output as supported by a previous study [62], and pathway inhibition as well as anti-tumor activity were also seen in the presence of genetic alterations within the SHH pathway previously shown to render tumors resistant to SMO inhibition [28].

Murine cells of origin for SHH-MB express high levels of the maintenance DNA methyltransferase DNMT1, and since expression of other DNA cytosine methyltransferases is virtually absent, we assume that consistent DNA hypomethylation effects across the genome of several SHH-MB tumor cell models are a direct consequence of the inhibition of DNMT1 function. On the gene expression level, the vast majority of changes at early and late time points after DNMT1 inhibition were mutually exclusive. As expected, this revealed a broad gene network that is directly or indirectly regulated by DNMT1 function. Of note, a well-curated set of positive SHH regulators scored among the top gene sets inhibited early upon DNMT1 inhibition, further supporting our findings that DNMT1 is an important regulator of SHH activation in SHH-MB.

While we note that DNMT1 by now is considered a common essential gene, inhibitors of DNMT1 function have an acceptable toxicity profile [26]. Furthermore, we are convinced that the involvement of DNMT1 in the SHH pathway and its potential targetability in SHH-MB independent from SHH-associated genetic alterations render it a promising target for these tumors. In this regard, similar to other potentially common essential cancer targets such as CDK4/6 and MEK kinases, further pharmacodynamic and toxicity considerations will need to be assessed for SHH-MB [4].

A further chemogenetic knockout CRISPR-Cas9 screen in SMB21 cells indicated that *Smoothened* knockout acts as a synergistic interactor of DNMT1 inhibition. In a similar context, previous RNAi screening of acute myeloid leukemia (AML) cell lines under 5-AzaC treatment identified genes of the SHH pathway as targetable molecular vulnerabilities, reporting for the first time synergism between SMO inhibitor and 5-AzaC when combined concurrently in vitro for AML [60]. These results in combination with the protein–protein interaction network showing that SMO and DNMT1 are functionally associated encouraged us to investigate combination treatment of SMO and DNMT1 inhibition. Combination treatment in vitro was significantly more effective in inhibiting tumor growth and SHH activation than either SMO and DNMT1 inhibitors alone, and this synergistic effect was seen in both murine and human cell models of SHH-MB sensitive to SMO inhibitors. Furthermore, combination therapy in an inducible mouse model of SHH-MB resulted in decreased proliferation indices as compared to both monotherapies at the end of drug treatment. While we did not observe a significantly longer overall survival for the combination group as compared to the SMO inhibitor monotherapy, we assume that this is due to the limited treatment regimen in our study, suggesting that continuous treatment schedules of concomitant drug application are necessary to result in improved survival statistics.

## Conclusions

This study provides a list of genetic dependencies in a faithful model system for SHH-MB. We show that DNMT1 is a promising therapeutic target in SHH-MBs that acts by blocking SHH activity downstream of genetic alterations known to confer resistance to SMO inhibitors. Furthermore, simultaneous inhibition of both DNMT1 and SMO acts synergistically to inhibit tumor growth in in vitro and in vivo models of SHH-MB. Thus, our data provide the basis for further investigation of DNMT1 inhibition both as monotherapy as well as in combination with SMO inhibitors as a new rationale to treat SHH-associated tumors.

### Supplementary Information

Additional file 1.

Additional file 2.

Additional file 3.

Additional file 4.

Additional file 5.

Additional file 6.

Additional file 7.

## Data Availability

Raw data from DNA methylation arrays and RNA sequencing is publicly available under GEO SuperSeries GSE269463. Original raw read counts and quality metrics from PoolQ for all CRISPR-Cas9 knockout screens can be found at figshare, https://doi.org/10.6084/m9.figshare.25992037.v1 [43]. All major code from this study, particularly all analyses of DNA methylation, RNA sequencing, and CRISPR screening data, are available on zenodo as a GitHub release, https://zenodo.org/doi/10.5281/zenodo.11547189 [42].

## Abbreviations

5-AzaC: 5-Azacytidine
AML: Acute myeloid leukemia
BAGEL: Bayesian analysis of gene essentiality
CC3: Cleaved caspase 3
CNS: Central nervous system
CRISPR: Clustered regularly interspaced short palindromic repeats
DNMT1: DNA methyltransferase 1
DNMT1-N, -M, -C: N terminal, middle, C terminal
EGL: External granule layer
FDR: False discovery rate
GCNP: Granule cell neuron precursor
GLI1/2: Glioma-associated oncogene homolog 1/2
H&E: Hematoxylin and eosin
IGL: Internal granule layer
i.p.: Intraperitoneal
KO: Knockout
LFB: Luxol fast blue
LFC: Log_2_ fold changes
MAGeCK: Model-based analysis of genome-wide CRISPR/Cas9 knockout
MB: Medulloblastoma
ML: Molecular layer
MLE: Maximum likelihood estimation method
MOI: Multiplicity of infection
NeuN: Neuronal nuclear protein
Pax6: Paired box 6
PCL: Purkinje cell layer
PCNA: Proliferating cell nuclear antigen
PDOX: Patient-derived xenografts organoids
PEG: Polyethylene glycol 300
PFA: Paraformaldehyde
PTCH1: Patched1
ROI: Region of interest
RRA: Robust ranking aggregation
SHH: Sonic hedgehog
SMO: Smoothened
SUFU: Suppressor of fused homology
TP53: Cellular tumor antigen p53
WM: White matter
WNT: Wingless
