# Supplementary material for: Genome-wide CRISPR-Cas9 knockout screens identify DNMT1 as a druggable dependency in sonic hedgehog medulloblastoma
Source: Acta Neuropathol Commun. 2024 Aug 7;12:125. doi: 10.1186/s40478-024-01831-x (PMC11304869; doi:10.1186/s40478-024-01831-x)
Supplement: Supplementary file 1 — Additional file 1. [file 40478_2024_1831_MOESM1_ESM.pdf]

Title

Genome-wide CRISPR-Cas9 knockout screens identify DNMT1 as a druggable dependency for sonic hedgehog medulloblastoma

Authors

Foteini Tsiami, Chiara Lago, Noemi Pozza, Federica Piccioni, Xuesong Zhao, Fabienne Lülsberg, David E. Root, Luca Tiberi, Marcel Kool, Jens Schittenhelm, Pratiti Bandopadhayay, Rosalind A. Segal, Ghazaleh Tabatabai, Daniel J. Merk*

*corresponding author

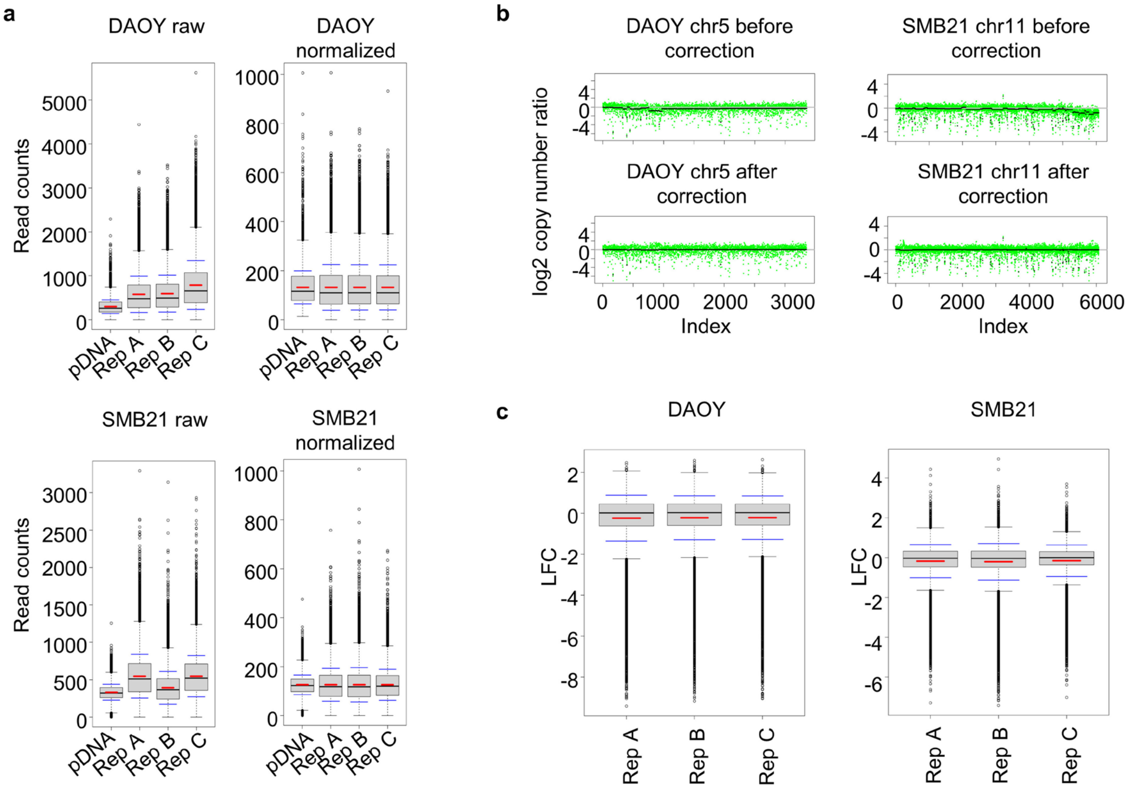

Fig. S1 Dropout screen analysis in DAOY and SMB21 cells using *CRISPRcleanR*. a Normalization of sgRNA read counts from reference plasmid (pDNA) and screen replicates (A-C) for DAOY (upper panel) and SMB21 cells (lower panel). **b** Representative copy number variation plots of chromosome 5 for DAOY (left) and chromosome 11 for SMB21 (right) cells, before (upper panel) and after correction (lower panel). **c** Box plots representing corrected gene log2 fold change of each screen replicate, as compared to the reference plasmid for DAOY (left) and SMB21 (right) cells.

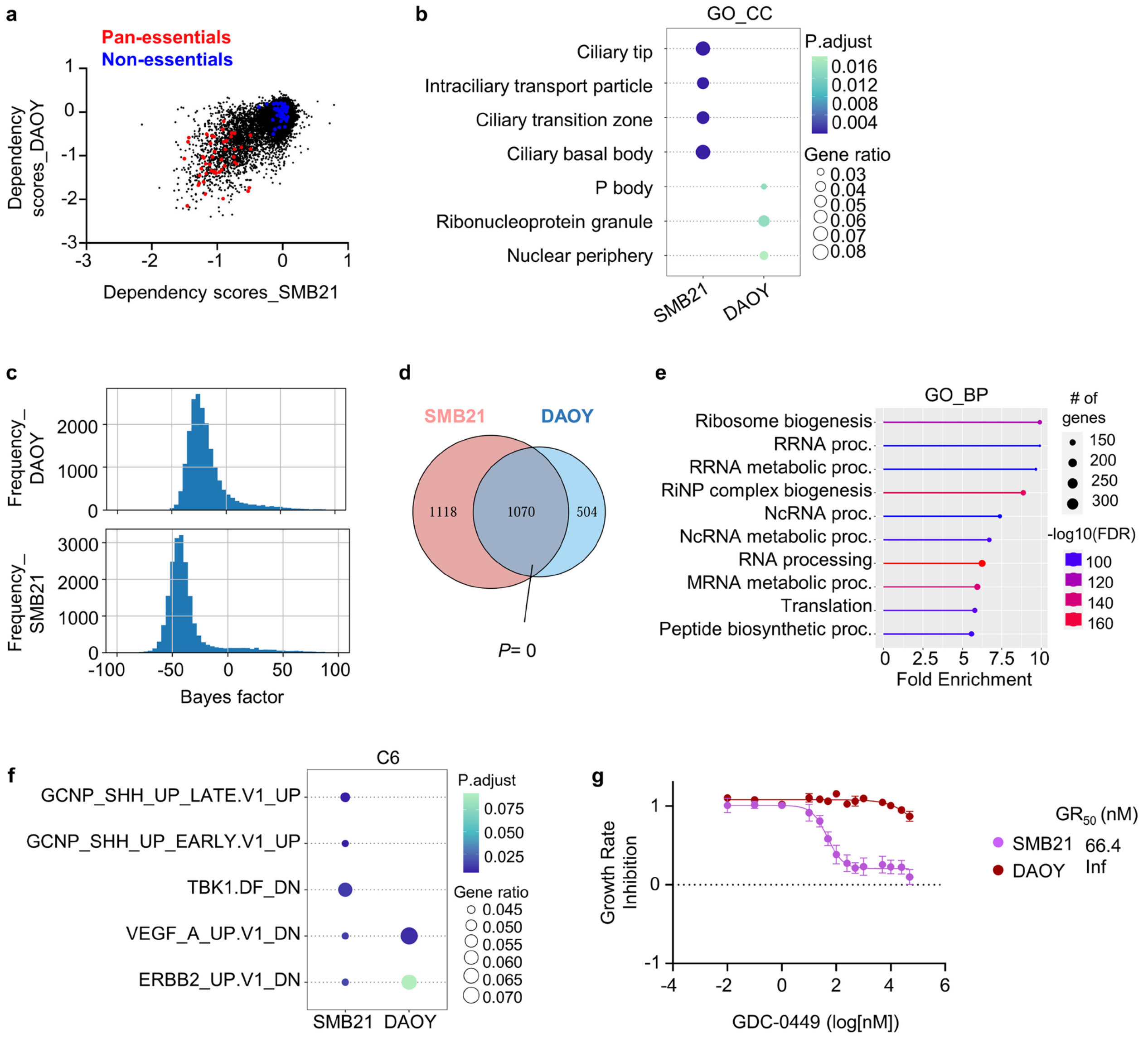

Fig. S2 Comparative analysis of SMB21 and DAOY dependencies. a Scatter plot illustrating dependency scores in SMB21 cells (x axis) and DAOY cells (y axis). Red data points indicate pan-species pan-essential genes, while blue data points non-essentials. **b** Dotplot illustrating gene ontology analysis of depleted genes in DAOY and SMB21 using cellular components terms (GO_CC). **c** Bar graphs demonstrating the distribution of Bayesian factors in DAOY (upper panel) and SMB21 (lower panel) cells. **d** Venn diagram of overlapping essentialities between SMB21 and DAOY cells at FDR<5%, as determined by MAGeCK-RRA and BAGEL2 algorithms. Statistics are derived from *SuperExactTest R* package. **e** Lollipop plot of the top 10 biological processes of shared essential genes by SMB21 and DAOY cells. FDR values are color-coded and number of genes enriched size-coded, as indicated on the side bars. **f** Dotplot illustrating top 5 C6 oncogenic gene sets enriched in DAOY and SMB21 context-specific dependencies. **g** Dose-dependent growth rate inhibition of SMB21 and DAOY cells treated with GDC-0449 for 72 hours. GR_{50} values are indicated per cell line, as calculated using the *GRmetrics* R package (n=4). Graph displays mean ± SD.

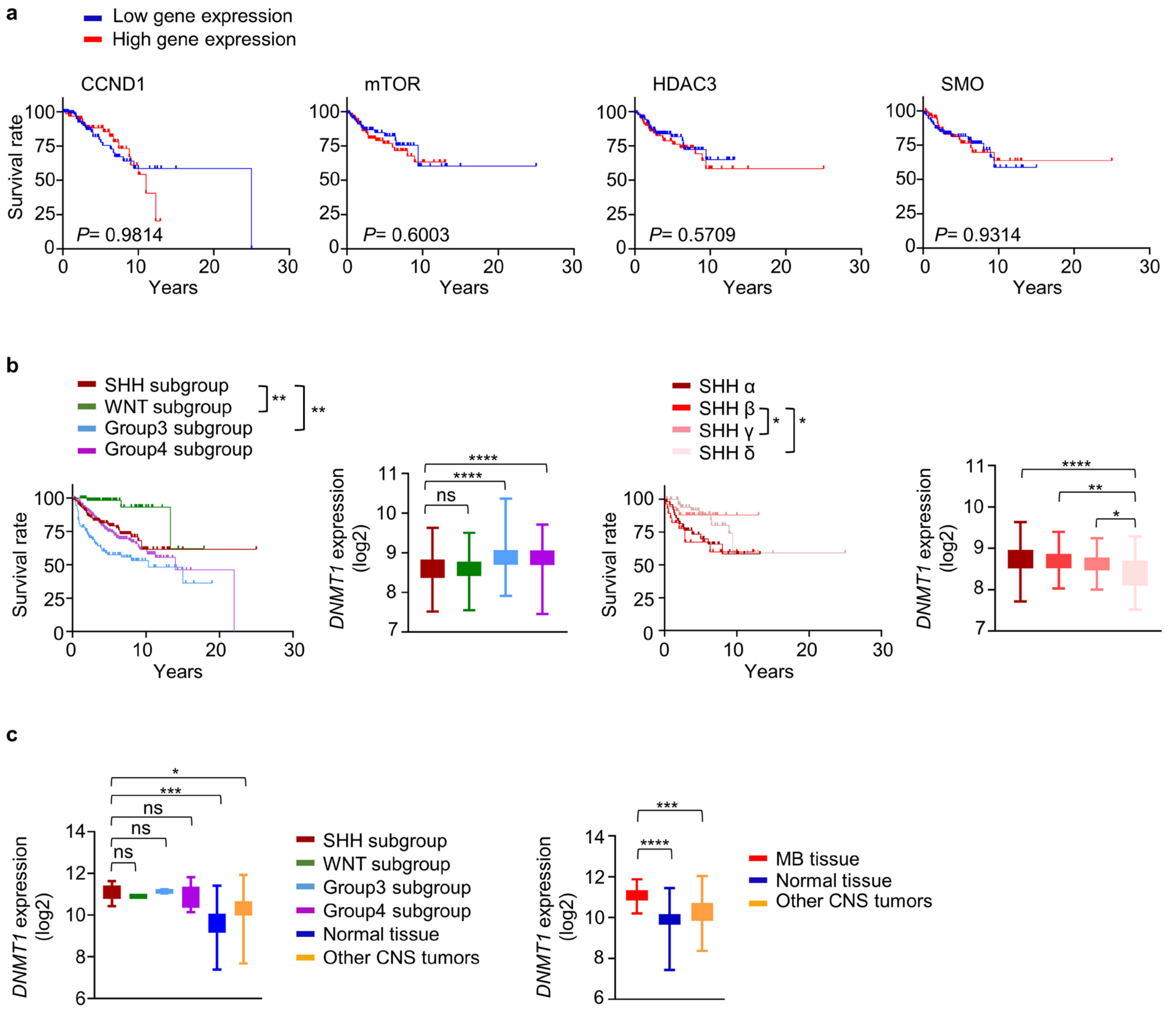

Fig. S3 Validation of selected genetic dependencies in MB patient cohorts. a Survival curves of SHH-MB patients with low and high expression of *CCND1*, *mTOR*, *HDAC3* and *SMO*, using Cavalli dataset. **b** Kaplan-Meier curves of MB patients and box plots illustrating *DNMT1* expression in patients assigned to four different MB subgroups (left panel) and to four SHH-MB subtypes (left panel), as determined from Cavalli dataset. **c** Box plots demonstrating *DNMT1* expression in patient-derived MB tissue, as well as other CNS tumors and normal brain tissue, using Gump (left plot) and Griesinger (right plot) datasets. Whiskers in all box plots represent minimum and maximum data point per group and one-way ANOVA, Tukey´s multiple comparisons test was performed. Significance in all survival curves is indicated using log rank (Mantel-Cox) test. All graphs display mean ± SD. *p ≤ 0.05, ** p ≤ 0.01, *** p ≤ 0.001, **** p ≤ 0.0001.

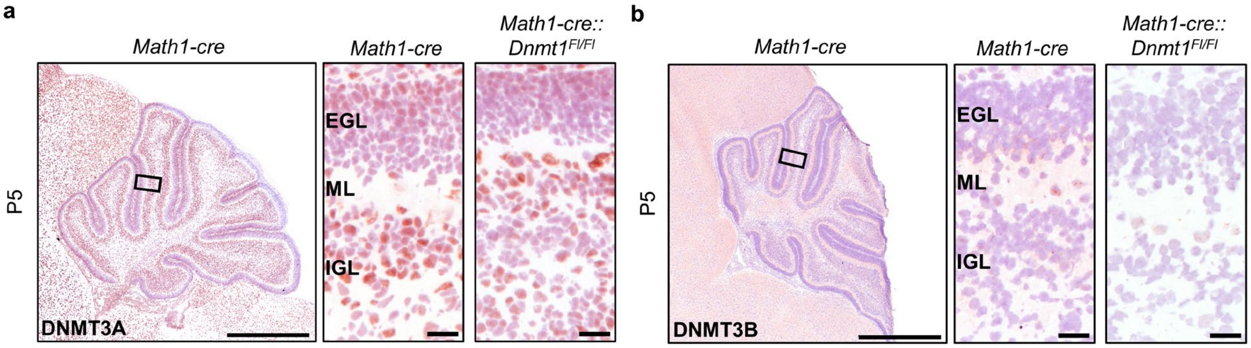

Fig. S4 DNMT3A and DNMT3B expression in murine cerebella. a Immunohistochemistry for DNMT3A in cerebella from *Math1-cre* and *Math1-cre::Dnmt1*$^{Fl/Fl}$ mice at p5. **b** Immunohistochemistry for DNMT3B in cerebella from *Math1-cre* and *Math1-cre::Dnmt1*$^{Fl/Fl}$ mice at p5. 4x magnification, scale bar, 500µm; 20x magnification, scale bar, 50µm. EGL, external granular layer; ML, molecular layer; IGL, internal granular layer.

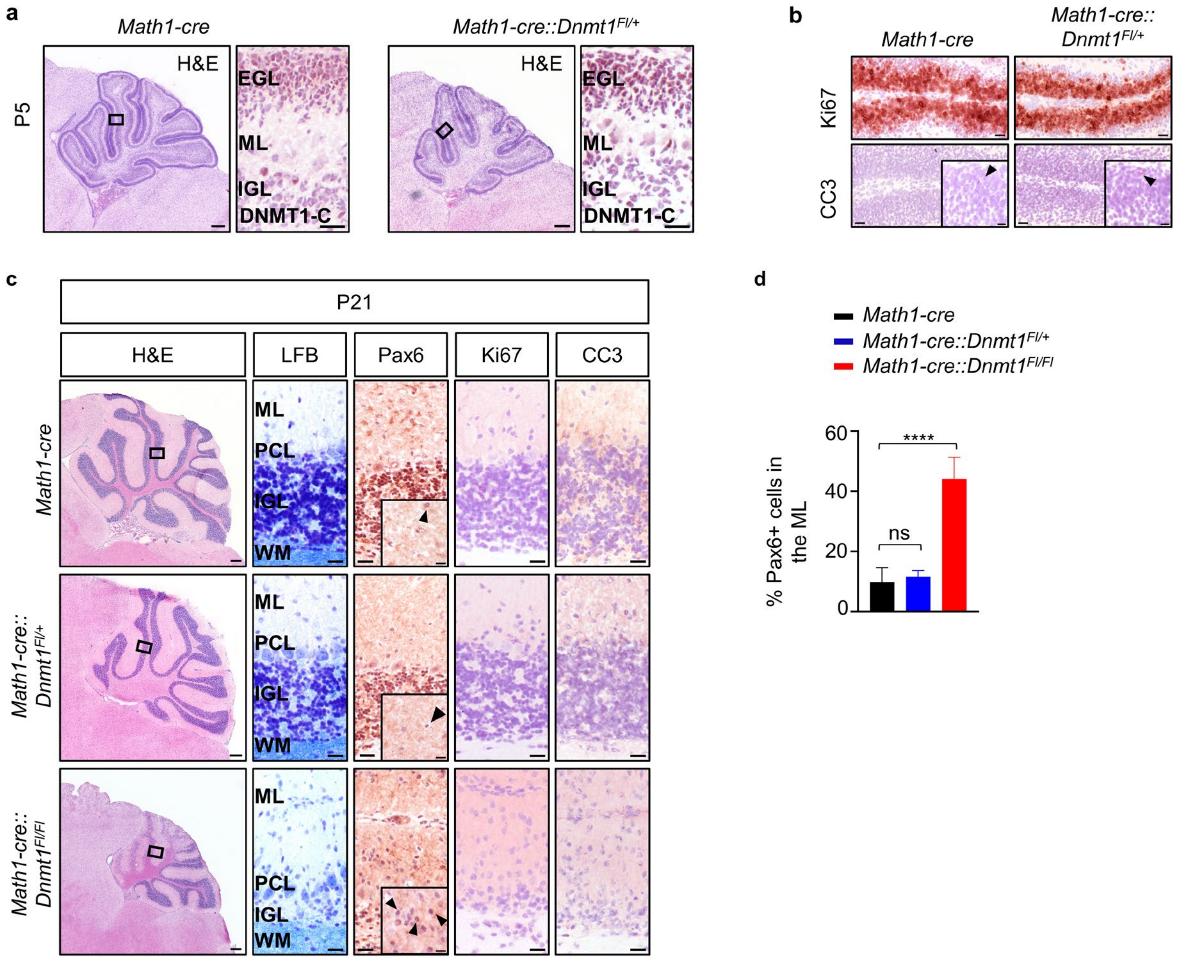

Fig. S5 Histological analysis of *Math1-cre::Dnmt1$^{Fl/+}$* and *Math1-cre::Dnmt1$^{Fl/Fl}$* mice. a Representative H&E stainings and immunohistochemistry for DNMT1-C of cerebella from *Math1-cre* and *Math1-cre::Dnmt1$^{Fl/+}$* mice at p5. **b** Immunohistochemistry for Ki67 and Cleaved Caspase 3 (CC3) in the EGL of cerebella from mice with indicated genotypes. Black arrowheads in the inset indicate CC3 positive cells. **c** Exemplary cerebellar sections of *Math1-cre*, *Math1-cre::Dnmt1$^{Fl/+}$* and *Math1-cre::Dnmt1$^{Fl/Fl}$* mice at P21 mice, stained with hematoxylin & eosin, luxol fast blue staining and antibodies specific for Pax6, Ki67 and cleaved caspase 3. Black arrowheads in the inset indicate Pax6-positive cells. **d** Quantification of Pax6-positive cells in the ML of cerebella mice with indicated genotypes at P21 (n=3, Fisher´s exact test). 4x magnification, scale bar, 500µm; 20x magnification, scale bar, 50µm, 40x magnification, scale bar; 20µm. EGL, external granular layer; ML, molecular layer; PCL, Purkinje cell layer; IGL, internal granular layer; WM, white matter. Graph displays mean ± SD. **** $p \leq 0.0001$.

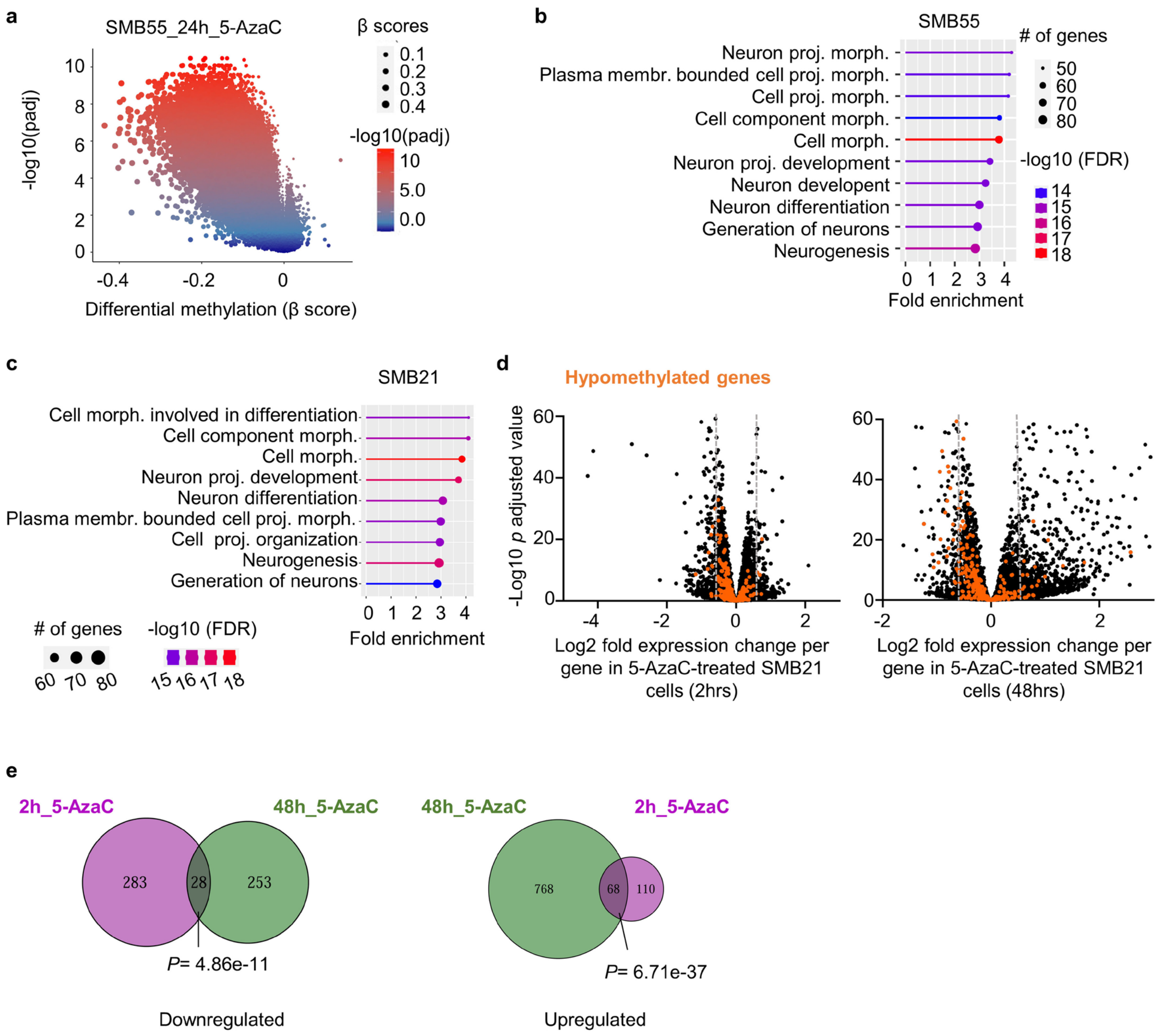

Fig. S6 DNMT1 inhibition alters global DNA methylation and gene expression in SHH-MB cells. a Volcano plots of differentially methylated probes in 5-AzaC-treated SMB55 cells compared to DMSO control-treated cells for 24 hours. P-adjusted values are color-coded and β scores size-coded, as indicated on the side bars. **b** Lollipop plot of the top biological processes of hypomethylated genes for SMB55 cells induced by 24 hours treatment with 5-AzaC. **c** Lollipop plot representing top enriched biological processes in 5-AzaC-treated SMB21 cells. **d** Volcano plots illustrating all differentially expressed genes in SMB21 cells after 2 hours (left) and 48 hours (right) treatment with 5-AzaC. Orange data points indicate hypomethylated genes in SMB21 cells. Dotted lines denote a threshold of $|\log_2\text{fold change}| \geq 0.58$. **e** Venn diagrams of overlapping downregulated (left) and upregulated genes (right) between 2h- and 48h-5-AzaC- treated SMB21 cells. Statistics are derived from *SuperExactTest* performed in *R.*

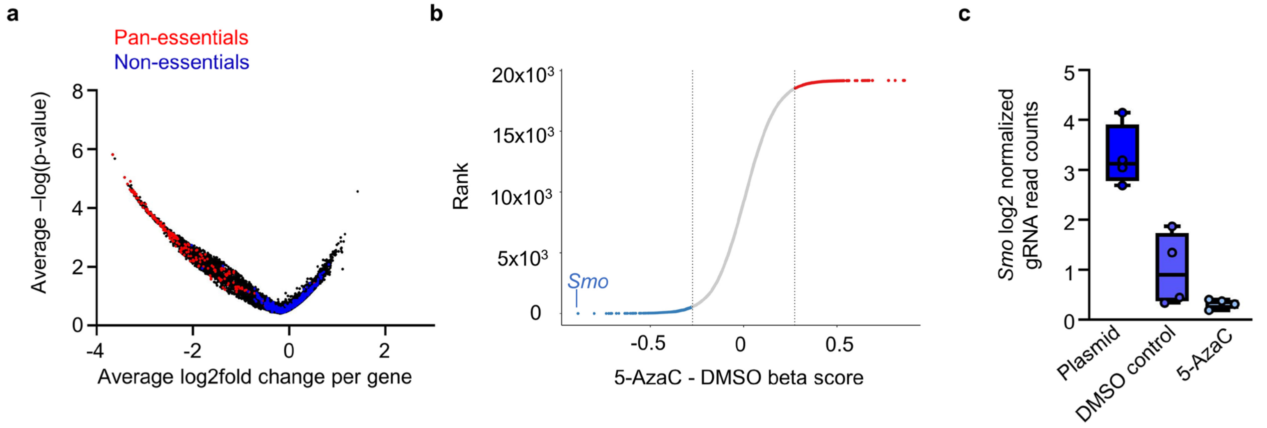

Fig. S7 Validation of a chemogenetic CRISPR-Cas9 knockout screen. **a** Volcano plot illustrating $\log_2$ fold change and associated *p* values per gene in DMSO control-treated SMB21 cells, when compared to the reference plasmid, using the hypergeometric distribution method. Red data points represent pan-essential genes and blue ones non-essential genes. **b** Rank plot of genes ranked by their differential β score, in which DMSO control´s β score is subtracted from the drug treatment´s. Dotted lines represent 1.5-fold standard deviation. **c** Box plots depicting the $\log_2$ normalized read counts per sgRNA targeting *Smo* in the reference plasmid, DMSO control and 5-AzaC arm. Data are shown as box plots with whiskers representing minimum and maximum data point per group.

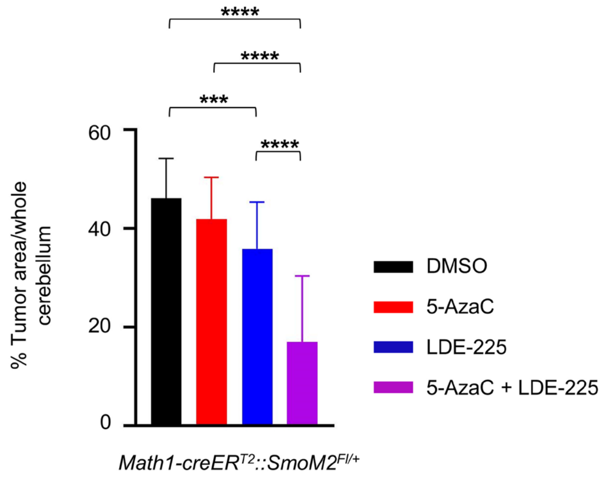

Fig. S8 Relative tumor area in *Math1-creER^{T2}::SmoM2$^{Fl/+}$* mice at P68. Percentage of tumor area to whole cerebellum in *Math1-creER^{T2}::SmoM2$^{Fl/+}$* mice from indicated groups at P68 (n=3, one-way ANOVA with Tukey´s multiple comparisons). Graph displays mean ± SD. *** $p \leq 0.001$, **** $p \leq 0.0001$.
